# Re-description, systematics and complete mitochondrial genome of *Philheliuscoreanus* (Shiraki, 1930) (Diptera, Syrphidae) in the Republic of Korea

**DOI:** 10.3897/BDJ.13.e146720

**Published:** 2025-04-07

**Authors:** Chan-Ouk Kim, Gyu-Dong Chang, Ho-Yeon Han, Jeong-Hun Song

**Affiliations:** 1 Department of Agricultural Biology, National Institute of Agricultural Sciences, Wanju, Republic of Korea Department of Agricultural Biology, National Institute of Agricultural Sciences Wanju Republic of Korea; 2 Yonsei University, Wonju, Republic of Korea Yonsei University Wonju Republic of Korea

**Keywords:** hoverfly, intraspecific variation, flower fly, *Xanthogramma*, phylogenetic analysis, Syrphini

## Abstract

**Background:**

The hoverfly *Philheliuscoreanus* (Shiraki, 1930) was first described, based on only Korean male specimens and subsequent descriptions of the female from Russia did not include discussions of phenotypic variation. Furthermore, full-length mitochondrial genome sequences for the genus are lacking.

**New information:**

To address these gaps, we here provide a diagnosis, re-description and mitochondrial genome of *Philheliuscoreanus* (Shiraki, 1930). We evaluated genitalic characters of both males and females with colour photographs and they showed intraspecific variation. There was significant variation in the yellow spots on the pleuron, particularly in females. After obtaining the complete mitochondrial genome of *P.coreanus*, we performed a phylogenetic analysis using Maximum Likelihood, based on 13 protein-coding genes, with a focus on relationships within the tribe Syrphini. Our results supported the monophyly of Syrphini, showing a sister-group relationship between *Philhelius* and *Doros* Meigen, 1822. Furthermore, the *Philhelius* + *Doros* clade was closely related to the *Chrysotoxum* + *Dideopsis* clade, with relatively high support. The newly-obtained mitochondrial genome of *P.coreanus* and high-resolution phylogenetic analysis provide essential resources for further analyses of the genus and relationships within Syrphini.

## Introduction

Hoverflies (Diptera, Syrphidae) are a large family of flies, with over 6,300 valid species recorded globally (Catalogue of Life, September 2024; www.catalogueoflife.org, Evenhuis and Pape (2024)). These insects are fascinating owing to their diverse morphological structures, feeding habits and habitats during their immature stages (Thompson and Rotheray 1998, Rotheray and Gilbert 2011, Inouye et al. 2015). Many species serve as pollinators in their adult stage, playing crucial roles in ecosystems, agriculture and forestry and are widely used as bioindicators (Sommaggio 1999, Ssymank et al. 2008, Hodgkiss et al. 2018, Cook et al. 2020, Dunn et al. 2020).

Recent phylogenetic studies divide Syrphidae into four subfamilies, Syrphinae, Pipizinae, Eristalinae and Microdontinae, of which only Eristalinae is paraphyletic (Mengual 2015, Moran et al. 2022, Mengual et al. 2023, Wong et al. 2023). Pipizinae, formerly treated as a tribe within Eristalinae, is now recognised as a distinct subfamily, sister to Syrphinae (Mengual et al. 2008, Mengual et al. 2023).

Within Syrphinae, three major tribes, Syrphini, Bacchini and Melanostomini, have been confirmed. Syrphini has been re-defined to include the former tribes Toxomerini and Paragini (Mengual et al. 2008, Mengual 2020, Mengual et al. 2023) and is consistently divided into two major lineages (Mengual et al. 2008, Mengual et al. 2018, Mengual et al. 2023).

Historically, the genus *Philhelius* Stephens, 1841, belonging to Syrphini, was frequently conflated with *Xanthogramma* Schiner, 1861. However, Evenhuis (2018) established that *Philhelius* is the valid senior synonym, thereby placing *Xanthogramma* in junior synonymy under it. Previously, *Philhelius* and *Olbiosyrphus* Mik, 1897 were distinguished, based on eye hairs; however, Vockeroth (1969) merged the latter into *Philhelius*. Phylogenetic studies have revealed that *Philhelius* and *Doros* Meigen, 1822 are sister taxa, closely related to *Chrysotoxum* Meigen, 1803 and *Epistrophe* Walker, 1852 (Mengual et al. 2008, Mengual et al. 2018, Mengual et al. 2023). However, mitogenome-based studies, with a differing taxon sampling and not including *Philhelius*, suggest slight differences in their relationships (Wong et al. 2023).

*Philheliuscoreanus* was first described by Shiraki (1930), based on Korean male specimens, with females described later by Violovitsh (1975) from Russian specimens, though without discussing their variability.

Many hoverfly species exhibit significant intraspecific morphological variation (Gilbert 1985, Milankov et al. 2009, Ricarte et al. 2020, Ballester-Torres et al. 2022, Aguado-Aranda et al. 2024), often leading to taxonomic challenges. Thus, we provide a detailed diagnosis and re-description of *P.coreanus*, incorporating genitalic characters of both sexes and highlighting intraspecific variation with colour photographs. Furthermore, we sequenced the complete mitochondrial genome of *P.coreanus*, providing the first full mitogenome for the genus and conducted a phylogenetic analysis based on 13 protein-coding genes (PCGs).

## Materials and methods

### Sample collection and morphological analysis

Most adult specimens of *P.coreanus* were collected using a hand-net, with a few collected by Malaise traps. Specimens of *P.coreanus* were identified, based on the original description (Shiraki 1930) and Violovitsh (1975). The morphological terminology mainly follow Cumming and Wood (2017); other genitalic terminology followed Dušek and Láska (1976) and Thompson (1999). In addition, Han and Norrbom (2005) and Kim and Han (2022) were followed for the following parameters: body length (from anterior margin of head excluding antenna to posterior margin of abdomen); wing length (from anterior margin of tegula to apex of vein R_4+5_); head ratio (head length/head height); face–head ratio (widest width of face in anterior view/width of head); eye ratio (shortest eye diameter/longest eye diameter); gena–eye ratio (genal height/longest eye diameter); antenna–head ratio (length of antenna/length of head); postpedicel–pedicel ratio (length of postpedicel/length of pedicel); arista–antenna ratio (length of arista/length of antenna excluding arista); wing ratio (wing length/wing width); wing–thorax ratio (wing length/thorax length); vein M ratio (distance along vein M between crossveins r-m and dm-cu/distance between crossveins r-m and bm-cu); and vein R_4+5_ ratio (distance along vein R_4+5_ between crossvein r-m and vein R_4+5_ apex/distance between crossvein r-m and basal node of vein R_4+5_).

Digital images from different focal planes (usually ≥ 50 per figure) were captured consecutively using a Fujifilm X-S1 camera (Tokyo, Japan) equipped with a Raynox DCR-240 macro-conversion lens. Photographs of the genitalia were taken using a Nikon D90 camera (Tokyo, Japan) mounted on an Olympus CX41 compound microscope (Tokyo, Japan). The captured images were then stacked using Helicon Focus (version 7.7.4, Helicon Soft, Ltd., Kharkiv, Ukraine).

The depositories of the examined specimens are as follows: Division of Biological Science and Technology, Yonsei University, Mirae Campus, Republic of Korea (**YSUW**); National Institute of Agricultural Sciences Insect Collection, Wanju, Republic of Korea (**NASIC**).

A single male specimen collected on 1 August 2018 from Mt. Taehwasan, Yeongwol-gun, Gangwon-do, Republic of Korea (37°07'03.3"N, 128°29'07.4"E) was used for mitochondrial genome sequencing. The specimen is deposited at the YSUW under voucher number YSUW210114673.

### Mitochondrial genome sequencing and analyses

The *P.coreanus* specimen was stored in a -23°C freezer and an abdomen sample was sent to Macrogen (Inc., Seoul, South Korea) for complete mitochondrial genome sequencing using the Illumina HiSeq platform (San Diego, CA, USA). A library was prepared using the Illumina TruSeq Nano DNA Kit. As raw data, 82,074,854 reads were produced, with a total base count of 12,393,302,954. After filtering using Trimmomatic v.0.36 (Bolger et al. 2014), 73,947,028 reads and 11,104,974,777 bases were obtained. The ratio of bases with a Phred quality score over 20 (Q20) was 99.15% and over 30 (Q30) was 97.01%. The clean reads were assembled using Geneious Prime (https://www.geneious.com) using the *Dideoideslatus* (Coquillett, 1898) mitogenome sequence (GenBank accession number: MZ315034; Li et al. (2023)) retrieved from GenBank (https://www.ncbi.nlm.nih.gov/genbank/; as of July 2024) as a reference. The complete mitogenome was annotated using the MITOS2 tool in the Galaxy platform (The Galaxy Community 2024; http://galaxyproject.org) and Geneious Prime (https://www.geneious.com). The base composition and codon usage were calculated using Geneious Prime and MEGA 11 (Tamura et al. 2021). A map of the mitogenome was drawn using OrganellarGenomeDRAW (https://chlorobox.mpimp-golm.mpg.de/OGDraw.html; Greiner et al. (2019)). Composition skew was analysed using the following formulae (Cameron 2014): AT skew = [A − T]/[A + T], GC skew = [G − C]/[G + C]. A+T-rich repeat regions were detected using Repeat Finder plugin v.1.0.1 in Geneious Prime v.2024.0.7 (https://www.geneious.com) with the following parameters: a minimum repeat length of 40 bp and maximum threshold of 10%.

### Phylogenetic analysis

For a phylogenetic analysis of Syrphini, 17 mitogenome sequences were obtained from GenBank, representing 17 species belonging to 17 genera. These include *Doros*, as well as *Chrysotoxum*, *Dasysyrphus* Enderlein, 1938, *Dideoides* Brunetti, 1908 and *Dideopsis* Matsumura, 1917, which are considered related to either *Philhelius* or *Doros* based on recent studies (Mengual et al. 2023, Wong et al. 2023). To confirm the relationship between Syrphini and other tribes belonging to Syrphinae, based on mitogenomes, one mitogenome was downloaded from each of the remaining tribes in Syrphinae, Bacchini and Melanostomini. Five species from the other three subfamilies, Pipizinae, Eristalinae and Microdontinae, were selected as outgroups. All of the samples included in analyses are described in Table 1.

For most species, 13 PCGs were included. However, for five species (*Pipizellaviduata* (Linnaeus, 1758), *Chrysotoxumbicinctum* (Linnaeus, 1758), *Bacchaelongata* (Fabricius, 1775), *Dorosdestillatorius* Mik, 1885 and *Milesiapendleburyi* Curran, 1928), data were not available for all 13 PCGs and between two (2,220 bp; *P.virduata*) and eight PCGs (6,945 bp; *D.destillatorius*) were analysed. Each PCG was aligned using MAFFT v. 7.490 (Katoh and Standley 2013) with the BLOSUM62 scoring matrix. All aligned PCGs were concatenated using Geneious Prime.

Best-fit partitioning schemes and substitution models of molecular evolution were determined using MixtureFinder v. 2.3.1, based on BIC (Bayesian Information Criterion) scores (Schwarz 1978), provided by the IQ-TREE web server (http://iqtree.cibiv.univie.ac.at/). The selected partitioning schemes and models were as follows: (i) GTR+F+G4 for *ND6*, (ii) GTR+F+I+G4 for *COX1, COX3, ND4* and *CYTB*, (iii) K3Pu+F+I+G4 for *ATP8* and *ND4L*, (iv) TIM+F+G4 for *ND3*, (v) TIM+F+I+G4 for *COX2, ATP6, ND5* and *ND1* and (vi) TVM+F+I+G4 for *ND2*.

A Maximum Likelihood (ML) tree was obtained using IQ TREE (Nguyen et al. 2015). Branch support was evaluated using 1,000 ultrafast bootstrap replicates (Minh et al. 2013) and 1,000 replications of the SH-aLRT branch test. The minimum correlation coefficient was set to 0.99.

## Taxon treatments

### Philhelius

Stephens, 1841

69379183-9560-57DD-8A60-BD2517B949BC

https://www.gbif.org/species/226376984

Philhelius Stephens, 1841Stephens (1841): 201. Type species: *Syrphusornatus* Meigen, 1822 = *Philheliuspedissequus* Harris, 1788.
Xanthogramma Schiner, 1861Schiner (1861): 318. Type species: *Syrphusornatus* Meigen, 1822 = *Philheliuspedissequus* Harris, 1758. Synonymised by Evenhuis (2018): 51–52.
Olbiosyrphus Mik, 1897Mik (1897): 66. Type species: *Syrphuslaetus* Fabricius, 1794. Synonymised by Vockeroth (1969): 90.
Syrphus ornatus Meigen, 1822

#### Diagnosis

**Figure 1.**
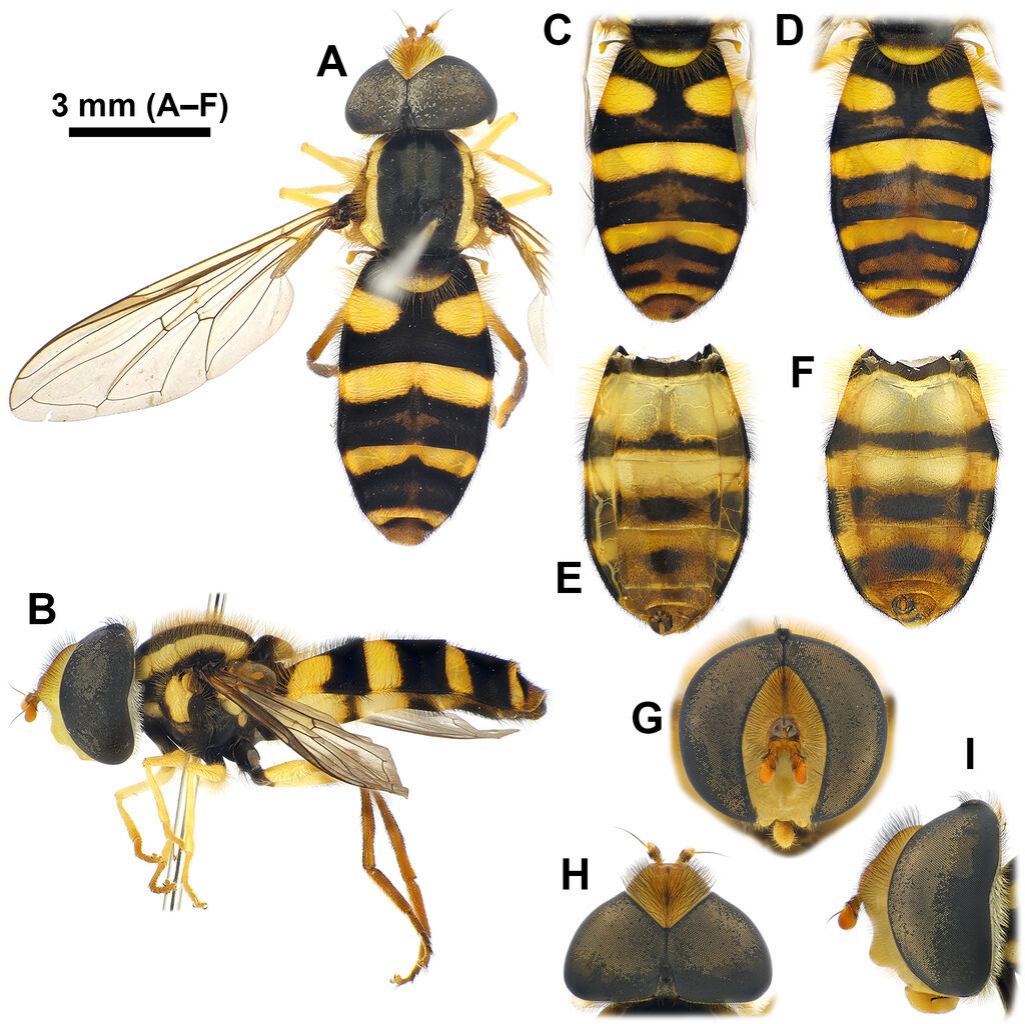
*Philheliuscoreanus*, males. **A, B** body, dorsal and lateral view; **C–F** abdomen: dorsal view (**C, D**); ventral view (**E, F**); **G–I** head: frontal view (**G**); dorsal view (**H**); lateral view (**I**).

The members of the genus *Philhelius* can be distinguished from other syrphid taxa by the following combination of characteristics [modified from Vockeroth (1969) and Thompson and Rotheray (1998)]: (1) postpronotum bare; (2) scutum predominantly black with distinct yellow lateral margins (Fig. 1A); (3) scutellum with the anterior half black and posterior half yellow approximately (Fig. 1A); (4) pleura predominantly black with yellow spots (Fig. 3); (5) dorsal and ventral katepisternal hair patches separated; (6) in male genitalia, postgonite relatively narrow and short, apico-dorsally with a long pointed upward hook (Fig. 4C); and (7) hypandrial lingula absent (Fig. 4C) and anteriorly with narrow concave corner in ventral view.

### Philhelius coreanus

(Shiraki, 1930)

FB3DDCB2-282C-51EE-A1B0-203B4740AE3C

https://www.gbif.org/species/11153940

*Xanthogrammacoreanum* Shiraki, 1930 Shiraki (1930): 403. Original description; Violovitsh (1975): 105. First females description; Violovitsh (1983): 34. Siberian syrphid identification key; Peck (1988): 51. Palaearctic catalogue; Huang et al. (1996): 159. Chinese checklist; Mutin and Barkalov (1999): 409. Russian Far East syrphid identification key; Han and Choi (2001): 74. Korean catalogue; Hua (2006): 73. Chinese checklist; Huo et al. (2007): 218. Chinese species identification key; Paek et al. (2010): 229. Korean checklist; Han et al. (2014): 49. Korean checklist; Barkalov and Mutin (2018) : 499. Russian checklist; Choi et al. (2018): 97. Korean catalogue; NIBR [National Institute of Biological Resources] (2019): 52. Korean checklist; Han et al. (2021): 443. Korean checklist.

#### Materials

- **Type status:** Other material. **Occurrence:** recordedBy: S.S. Euo, C.O. Kim, J.H. Choi; individualCount: 1; sex: female; lifeStage: adult; occurrenceID: F5BDA6D1-8242-5364-80E7-D0B53C17655C; **Taxon:** scientificName: Philheliuscoreanus; **Location:** country: South Korea; stateProvince: Chungcheongbuk-do; locality: Mt. Sambongsan, Goja-ri, Sangchon-myeon, Yeongdong-gun; verbatimLocality: 2018-07-25; verbatimCoordinates: 35°26'50"N 127°40'10"E; **Identification:** identifiedBy: Chan-Ouk Kim; dateIdentified: 2024; **Event:** eventDate: 2018-07-25; **Record Level:** language: en; institutionID: YSUW; collectionCode: Insects; basisOfRecord: PreservedSpecimen
- **Type status:** Other material. **Occurrence:** recordedBy: S.S. Euo, C.O. Kim, J.H. Choi; individualCount: 1; sex: female; lifeStage: adult; occurrenceID: 50604E32-6101-578A-8BD5-640D7DF9B872; **Taxon:** scientificName: Philheliuscoreanus; **Location:** country: South Korea; stateProvince: Chungcheongbuk-do; locality: Mt. Sambongsan, Goja-ri, Sangchon-myeon, Yeongdong-gun; verbatimLocality: 2019-06-25; verbatimCoordinates: 35°26'50"N 127°40'10"E; **Identification:** identifiedBy: Chan-Ouk Kim; dateIdentified: 2024; **Event:** eventDate: 2019-06-25; **Record Level:** language: en; institutionID: YSUW; collectionCode: Insects; basisOfRecord: PreservedSpecimen
- **Type status:** Other material. **Occurrence:** recordedBy: S.S. Euo, C.O. Kim, J.H. Choi; individualCount: 1; sex: female; lifeStage: adult; occurrenceID: 7AA18A10-3D49-5817-BA58-5F89DDF89E71; **Taxon:** scientificName: Philheliuscoreanus; **Location:** country: South Korea; stateProvince: Chungcheongbuk-do; locality: Mt. Gakhosan, Jodong-ri, Yonghwa-myeon, Yeongdong-gun; verbatimLocality: 2018-07-25; verbatimCoordinates: 36°04'08.6"N 127°50'07.1"E; **Identification:** identifiedBy: Chan-Ouk Kim; dateIdentified: 2024; **Event:** eventDate: 2018-07-25; **Record Level:** language: en; institutionID: YSUW; collectionCode: Insects; basisOfRecord: PreservedSpecimen
- **Type status:** Other material. **Occurrence:** recordedBy: S.W. Suk et al.; individualCount: 1; sex: male; lifeStage: adult; occurrenceID: 145D87AA-1129-5FA0-97FF-336611B5C1DE; **Taxon:** scientificName: Philheliuscoreanus; **Location:** country: South Korea; stateProvince: Gyeongsangbuk-do; locality: Mt. Cheongnyangsan, Cheukyungbong, Myeongho-myeon, Bonghwa-gun; verbatimLocality: 2012-06-13; verbatimElevation: 845 m; verbatimCoordinates: 36°46'27"N 128°54'46"E; **Identification:** identifiedBy: Chan-Ouk Kim; dateIdentified: 2024; **Event:** eventDate: 2012-06-13; **Record Level:** language: en; institutionID: YSUW; collectionCode: Insects; basisOfRecord: PreservedSpecimen
- **Type status:** Other material. **Occurrence:** recordedBy: S.W. Suk et al.; individualCount: 1; sex: female; lifeStage: adult; occurrenceID: 12518C61-B89B-5D87-B781-E07CCAC79C0F; **Taxon:** scientificName: Philheliuscoreanus; **Location:** country: South Korea; stateProvince: Gyeongsangbuk-do; locality: Mt. Cheongnyangsan, Cheukyungbong, Myeongho-myeon, Bonghwa-gun; verbatimLocality: 2012-06-13; verbatimElevation: 845 m; verbatimCoordinates: 36°46'27"N 128°54'46"E; **Identification:** identifiedBy: Chan-Ouk Kim; dateIdentified: 2024; **Event:** eventDate: 2012-06-13; **Record Level:** language: en; institutionID: YSUW; collectionCode: Insects; basisOfRecord: PreservedSpecimen
- **Type status:** Other material. **Occurrence:** recordedBy: Y.B. Lee, S.S. Euo, S.H. Jeong; individualCount: 1; sex: female; lifeStage: adult; occurrenceID: B38C44D0-07F4-50C4-A193-6107079E577F; **Taxon:** scientificName: Philheliuscoreanus; **Location:** country: South Korea; stateProvince: Gyeongsangbuk-do; locality: Mt. Palgongsan, from Hanti Reststop to Pagyebong, Bugye-myeon, Gunwi-gun; verbatimLocality: 2014-06-27; verbatimElevation: 1086 m; verbatimCoordinates: 36°0'60"N 128°39'25"E; **Identification:** identifiedBy: Chan-Ouk Kim; dateIdentified: 2024; **Event:** eventDate: 2014-06-27; **Record Level:** language: en; institutionID: YSUW; collectionCode: Insects; basisOfRecord: PreservedSpecimen
- **Type status:** Other material. **Occurrence:** recordedBy: S.S. Euo, C.O. Kim, J.H. Choi; individualCount: 2; sex: female; lifeStage: adult; occurrenceID: DE8B7316-F5F6-5983-A9F4-AE085FDDDF5B; **Taxon:** scientificName: Philheliuscoreanus; **Location:** country: South Korea; stateProvince: Gangwon-do; locality: Mt. Chiaksan, from Gangrim4-ri to Cheonjibong, Anheung-myeon, Hoengseong-gun; verbatimLocality: 2018-07-17; verbatimElevation: 1086 m; verbatimCoordinates: 37°23'51"N 128°05'23"E; **Identification:** identifiedBy: Chan-Ouk Kim; dateIdentified: 2024; **Event:** eventDate: 2018-07-17; **Record Level:** language: en; institutionID: YSUW; collectionCode: Insects; basisOfRecord: PreservedSpecimen
- **Type status:** Other material. **Occurrence:** recordedBy: D.S. Choi, S.K. Kim, D.S. Kang; individualCount: 1; sex: male; lifeStage: adult; occurrenceID: 7673A62D-45DB-51DC-A247-89E64B060014; **Taxon:** scientificName: Philheliuscoreanus; **Location:** country: South Korea; stateProvince: Gangwon-do; locality: Mt. Cheongtaesan, Sapgyo-ri, Dunnae-myeon, Hoengseong-gun; verbatimLocality: 2001-07-07; verbatimCoordinates: 37°30'40"N 128°18'01"E; **Identification:** identifiedBy: Chan-Ouk Kim; dateIdentified: 2024; **Event:** eventDate: 2001-07-07; **Record Level:** language: en; institutionID: YSUW; collectionCode: Insects; basisOfRecord: PreservedSpecimen
- **Type status:** Other material. **Occurrence:** recordedBy: S.S. Euo, C.O. Kim, J.H. Choi; individualCount: 5; sex: female; lifeStage: adult; occurrenceID: BEEF8181-A17E-5232-901C-D39B343FD61E; **Taxon:** scientificName: Philheliuscoreanus; **Location:** country: South Korea; stateProvince: Gangwon-do; locality: Woldoon-gol, Gwangwon-ri, Nae-myeon, Hongcheon-gun; verbatimLocality: 2018-07-26; verbatimCoordinates: 37°50'52"N 128°25'24"E; **Identification:** identifiedBy: Chan-Ouk Kim; dateIdentified: 2024; **Event:** eventDate: 2018-07-26; **Record Level:** language: en; institutionID: YSUW; collectionCode: Insects; basisOfRecord: PreservedSpecimen
- **Type status:** Other material. **Occurrence:** recordedBy: Y.B. Lee et al.; individualCount: 2; sex: female; lifeStage: adult; occurrenceID: 0B3348A2-DB6C-5251-BF18-D5EC26849061; **Taxon:** scientificName: Philheliuscoreanus; **Location:** country: South Korea; stateProvince: Gangwon-do; locality: Woldoon-gol, Gwangwon-ri, Nae-myeon, Hongcheon-gun; verbatimLocality: 2019-08-06; **Identification:** identifiedBy: Chan-Ouk Kim; dateIdentified: 2024; **Event:** eventDate: 2019-08-06; **Record Level:** language: en; institutionID: YSUW; collectionCode: Insects; basisOfRecord: PreservedSpecimen
- **Type status:** Other material. **Occurrence:** recordedBy: S.S. Euo, C.O. Kim,; individualCount: 3; sex: female; lifeStage: adult; occurrenceID: DEB2A384-2C1E-5068-A131-225BA9663866; **Taxon:** scientificName: Philheliuscoreanus; **Location:** country: South Korea; stateProvince: Gangwon-do; locality: Woldoon-gol, Gwangwon-ri, Nae-myeon, Hongcheon-gun; verbatimLocality: 2019-08-12; **Identification:** identifiedBy: Chan-Ouk Kim; dateIdentified: 2024; **Event:** eventDate: 2019-08-12; **Record Level:** language: en; institutionID: YSUW; collectionCode: Insects; basisOfRecord: PreservedSpecimen
- **Type status:** Other material. **Occurrence:** recordedBy: J.W. Lee; individualCount: 1; sex: female; lifeStage: adult; occurrenceID: C611A274-F152-5148-9BCC-46D4CB0F942A; **Taxon:** scientificName: Philheliuscoreanus; **Location:** country: South Korea; stateProvince: Gangwon-do; locality: Mt. Gyebangsan, Nae-myeon, Hongcheon-gun,; verbatimLocality: 2013-06-24/07-19; **Identification:** identifiedBy: Chan-Ouk Kim; dateIdentified: 2024; **Event:** eventDate: 2013-06-24/07-19; **Record Level:** language: en; institutionID: YSUW; collectionCode: Insects; basisOfRecord: PreservedSpecimen
- **Type status:** Other material. **Occurrence:** recordedBy: H.Y. Han et al.; individualCount: 1; sex: male; lifeStage: adult; occurrenceID: 0436DFE6-CD98-5926-8804-871D225E793F; **Taxon:** scientificName: Philheliuscoreanus; **Location:** country: South Korea; stateProvince: Gangwon-do; locality: Mt. Bangtaesan, from Bangtae Recreational forest, Bangdong-ri, Girin-myeon, Inje-gun; verbatimLocality: 2015-06-19; verbatimCoordinates: 37°55'46"N 128°23'18"E; **Identification:** identifiedBy: Chan-Ouk Kim; dateIdentified: 2024; **Event:** eventDate: 2015-06-19; **Record Level:** language: en; institutionID: YSUW; collectionCode: Insects; basisOfRecord: PreservedSpecimen
- **Type status:** Other material. **Occurrence:** recordedBy: S.S. Euo, C.O. Kim, J.H. Choi; individualCount: 1; sex: female; lifeStage: adult; occurrenceID: ABCBE250-1DA3-56E8-AC15-DC422DC4DF09; **Taxon:** scientificName: Philheliuscoreanus; **Location:** country: South Korea; stateProvince: Gangwon-do; locality: Mt. Maebongsan, Seo-ri, Girin-myeon, Inje-gun; verbatimLocality: 2017-07-06; verbatimCoordinates: 37°56'43.1"N 128°13'40"E; **Identification:** identifiedBy: Chan-Ouk Kim; dateIdentified: 2024; **Event:** eventDate: 2017-07-06; **Record Level:** language: en; institutionID: YSUW; collectionCode: Insects; basisOfRecord: PreservedSpecimen
- **Type status:** Other material. **Occurrence:** recordedBy: H.Y. Han, Y.B. Lee, S.H. Jeong; individualCount: 1; sex: male; lifeStage: adult; occurrenceID: 4C38F5A2-496D-5AE1-99A5-6A050982620C; **Taxon:** scientificName: Philheliuscoreanus; **Location:** country: South Korea; stateProvince: Gangwon-do; locality: Mt. Hanseoksan, Deokjeok-ri, Inje-eup, Inje-gun; verbatimLocality: 2016-06-10; verbatimCoordinates: 38°3'14"N 128°14'40"E; **Identification:** identifiedBy: Chan-Ouk Kim; dateIdentified: 2024; **Event:** eventDate: 2016-06-10; **Record Level:** language: en; institutionID: YSUW; collectionCode: Insects; basisOfRecord: PreservedSpecimen
- **Type status:** Other material. **Occurrence:** recordedBy: H.Y. Han et al.; individualCount: 2; sex: male; lifeStage: adult; occurrenceID: F702431E-38F1-5582-86B6-0967AE6EFA40; **Taxon:** scientificName: Philheliuscoreanus; **Location:** country: South Korea; stateProvince: Gangwon-do; locality: Mt. Mindungsan, Yupyeong-ri, Nam-myeon, Jeongseon-gun; verbatimLocality: 2008-07-04; verbatimCoordinates: 37°16'10"N 128°46'49"E; **Identification:** identifiedBy: Chan-Ouk Kim; dateIdentified: 2024; **Event:** eventDate: 2008-07-04; **Record Level:** language: en; institutionID: YSUW; collectionCode: Insects; basisOfRecord: PreservedSpecimen
- **Type status:** Other material. **Occurrence:** recordedBy: H.Y. Han et al.; individualCount: 1; sex: female; lifeStage: adult; occurrenceID: C5770032-E21C-59C0-A92E-DE4819847328; **Taxon:** scientificName: Philheliuscoreanus; **Location:** country: South Korea; stateProvince: Gangwon-do; locality: Mt. Mindungsan, Yupyeong-ri, Nam-myeon, Jeongseon-gun; verbatimLocality: 2008-07-04; verbatimCoordinates: 37°16'10"N 128°46'49"E; **Identification:** identifiedBy: Chan-Ouk Kim; dateIdentified: 2024; **Event:** eventDate: 2008-07-04; **Record Level:** language: en; institutionID: YSUW; collectionCode: Insects; basisOfRecord: PreservedSpecimen
- **Type status:** Other material. **Occurrence:** recordedBy: S.W. Suk, D.J. Cha, Y.B. Lee; individualCount: 1; sex: male; lifeStage: adult; occurrenceID: 41FB3AA8-1A51-5A8D-853F-3404B399F11F; **Taxon:** scientificName: Philheliuscoreanus; **Location:** country: South Korea; stateProvince: Gangwon-do; locality: Mt. Mindungsan, Yupyeong-ri, Nam-myeon, Jeongseon-gun; verbatimLocality: 2008-08-29; **Identification:** identifiedBy: Chan-Ouk Kim; dateIdentified: 2024; **Event:** eventDate: 2008-08-29; **Record Level:** language: en; institutionID: YSUW; collectionCode: Insects; basisOfRecord: PreservedSpecimen
- **Type status:** Other material. **Occurrence:** recordedBy: H.Y. Han et al.; individualCount: 1; sex: male; lifeStage: adult; occurrenceID: 35078B9C-0FAB-5133-B553-7BEE598980D7; **Taxon:** scientificName: Philheliuscoreanus; **Location:** country: South Korea; stateProvince: Gangwon-do; locality: Mt. Mindungsan, Yupyeong-ri, Nam-myeon, Jeongseon-gun; verbatimLocality: 2011-06-17; **Identification:** identifiedBy: Chan-Ouk Kim; dateIdentified: 2024; **Event:** eventDate: 2011-06-17; **Record Level:** language: en; institutionID: YSUW; collectionCode: Insects; basisOfRecord: PreservedSpecimen
- **Type status:** Other material. **Occurrence:** recordedBy: S.W. Suk, H.S. Lee, D.H. Kim; individualCount: 2; sex: male; lifeStage: adult; occurrenceID: 6BC5510D-8DC1-5A9F-970E-074D13588288; **Taxon:** scientificName: Philheliuscoreanus; **Location:** country: South Korea; stateProvince: Gangwon-do; locality: Mt. Mindungsan, Yupyeong-ri, Nam-myeon, Jeongseon-gun; verbatimLocality: 2012-06-18; **Identification:** identifiedBy: Chan-Ouk Kim; dateIdentified: 2024; **Event:** eventDate: 2012-06-18; **Record Level:** language: en; institutionID: YSUW; collectionCode: Insects; basisOfRecord: PreservedSpecimen
- **Type status:** Other material. **Occurrence:** recordedBy: S.W. Suk, H.S. Lee, D.H. Kim; individualCount: 1; sex: female; lifeStage: adult; occurrenceID: 07A6209B-2BF7-59BC-AA40-9AC217683B5A; **Taxon:** scientificName: Philheliuscoreanus; **Location:** country: South Korea; stateProvince: Gangwon-do; locality: Mt. Mindungsan, Yupyeong-ri, Nam-myeon, Jeongseon-gun; verbatimLocality: 2012-06-18; **Identification:** identifiedBy: Chan-Ouk Kim; dateIdentified: 2024; **Event:** eventDate: 2012-06-18; **Record Level:** language: en; institutionID: YSUW; collectionCode: Insects; basisOfRecord: PreservedSpecimen
- **Type status:** Other material. **Occurrence:** recordedBy: H.Y. Han et al.; individualCount: 2; sex: female; lifeStage: adult; occurrenceID: 36478B8B-3987-56C0-8BF3-D59C4D88D0DD; **Taxon:** scientificName: Philheliuscoreanus; **Location:** country: South Korea; stateProvince: Gangwon-do; locality: Mt. Mindungsan, Yupyeong-ri, Nam-myeon, Jeongseon-gun; verbatimLocality: 2012-06-28; **Identification:** identifiedBy: Chan-Ouk Kim; dateIdentified: 2024; **Event:** eventDate: 2012-06-28; **Record Level:** language: en; institutionID: YSUW; collectionCode: Insects; basisOfRecord: PreservedSpecimen
- **Type status:** Other material. **Occurrence:** recordedBy: Y.B. Lee, S.S. Euo, S.H. Jeong; individualCount: 2; sex: female; lifeStage: adult; occurrenceID: 2C0B27B9-DC84-5800-A6B0-8E95DC701E6B; **Taxon:** scientificName: Philheliuscoreanus; **Location:** country: South Korea; stateProvince: Gangwon-do; locality: Mt. Mindungsan, Yupyeong-ri, Nam-myeon, Jeongseon-gun; verbatimLocality: 2015-08-17; **Identification:** identifiedBy: Chan-Ouk Kim; dateIdentified: 2024; **Event:** eventDate: 2015-08-17; **Record Level:** language: en; institutionID: YSUW; collectionCode: Insects; basisOfRecord: PreservedSpecimen
- **Type status:** Other material. **Occurrence:** recordedBy: H.Y. Han et al.; individualCount: 4; sex: male; lifeStage: adult; occurrenceID: 1147D1E3-44BC-58EA-9475-766239D81FA9; **Taxon:** scientificName: Philheliuscoreanus; **Location:** country: South Korea; stateProvince: Gangwon-do; locality: Mt. Mindungsan, Yupyeong-ri, Nam-myeon, Jeongseon-gun; verbatimLocality: 2016-07-10; **Identification:** identifiedBy: Chan-Ouk Kim; dateIdentified: 2024; **Event:** eventDate: 2016-07-10; **Record Level:** language: en; institutionID: YSUW; collectionCode: Insects; basisOfRecord: PreservedSpecimen
- **Type status:** Other material. **Occurrence:** recordedBy: H.Y. Han et al.; individualCount: 5; sex: female; lifeStage: adult; occurrenceID: 844B7620-3395-5612-9745-7643390AABDA; **Taxon:** scientificName: Philheliuscoreanus; **Location:** country: South Korea; stateProvince: Gangwon-do; locality: Mt. Mindungsan, Yupyeong-ri, Nam-myeon, Jeongseon-gun; verbatimLocality: 2016-07-10; **Identification:** identifiedBy: Chan-Ouk Kim; dateIdentified: 2024; **Event:** eventDate: 2016-07-10; **Record Level:** language: en; institutionID: YSUW; collectionCode: Insects; basisOfRecord: PreservedSpecimen
- **Type status:** Other material. **Occurrence:** recordedBy: S.S. Euo, W.R. Ha, J.H. Choi; individualCount: 1; sex: male; lifeStage: adult; occurrenceID: E07CFFEE-D40B-5457-A563-48206C82371C; **Taxon:** scientificName: Philheliuscoreanus; **Location:** country: South Korea; stateProvince: Gangwon-do; locality: Mt. Mindungsan, Yupyeong-ri, Nam-myeon, Jeongseon-gun; verbatimLocality: 2017-07-12; **Identification:** identifiedBy: Chan-Ouk Kim; dateIdentified: 2024; **Event:** eventDate: 2017-07-12; **Record Level:** language: en; institutionID: YSUW; collectionCode: Insects; basisOfRecord: PreservedSpecimen
- **Type status:** Other material. **Occurrence:** recordedBy: S.S. Euo, W.R. Ha, J.H. Choi; individualCount: 6; sex: female; lifeStage: adult; occurrenceID: B0AE5056-A97B-5E4B-A350-A7138CF2A16C; **Taxon:** scientificName: Philheliuscoreanus; **Location:** country: South Korea; stateProvince: Gangwon-do; locality: Mt. Mindungsan, Yupyeong-ri, Nam-myeon, Jeongseon-gun; verbatimLocality: 2017-07-12; **Identification:** identifiedBy: Chan-Ouk Kim; dateIdentified: 2024; **Event:** eventDate: 2017-07-12; **Record Level:** language: en; institutionID: YSUW; collectionCode: Insects; basisOfRecord: PreservedSpecimen
- **Type status:** Other material. **Occurrence:** recordedBy: S.S. Euo et al.; individualCount: 6; sex: female; lifeStage: adult; occurrenceID: 225E2C3A-D914-5EE5-889F-40CC844F4230; **Taxon:** scientificName: Philheliuscoreanus; **Location:** country: South Korea; stateProvince: Gangwon-do; locality: Mt. Mindungsan, Yupyeong-ri, Nam-myeon, Jeongseon-gun; verbatimLocality: 2017-07-26; **Identification:** identifiedBy: Chan-Ouk Kim; dateIdentified: 2024; **Event:** eventDate: 2017-07-26; **Record Level:** language: en; institutionID: YSUW; collectionCode: Insects; basisOfRecord: PreservedSpecimen
- **Type status:** Other material. **Occurrence:** recordedBy: S.S. Euo, C.O. Kim, J.H. Choi; individualCount: 8; sex: female; lifeStage: adult; occurrenceID: 44FDB38C-9A2F-5DD1-89D7-239A75AB0A2A; **Taxon:** scientificName: Philheliuscoreanus; **Location:** country: South Korea; stateProvince: Gangwon-do; locality: Mt. Mindungsan, Yupyeong-ri, Nam-myeon, Jeongseon-gun; verbatimLocality: 2018-07-12; **Identification:** identifiedBy: Chan-Ouk Kim; dateIdentified: 2024; **Event:** eventDate: 2018-07-12; **Record Level:** language: en; institutionID: YSUW; collectionCode: Insects; basisOfRecord: PreservedSpecimen
- **Type status:** Other material. **Occurrence:** recordedBy: S.S. Euo et al.; individualCount: 1; sex: female; lifeStage: adult; occurrenceID: DC4C9984-A0C3-529D-9A85-C7244D94D410; **Taxon:** scientificName: Philheliuscoreanus; **Location:** country: South Korea; stateProvince: Gangwon-do; locality: Mt. Geunsan, Geunsan-dong, Samcheok-si,; verbatimLocality: 2017-07-27; verbatimCoordinates: 37°24'48"N 129°08'29"E; **Identification:** identifiedBy: Chan-Ouk Kim; dateIdentified: 2024; **Event:** eventDate: 2017-07-27; **Record Level:** language: en; institutionID: YSUW; collectionCode: Insects; basisOfRecord: PreservedSpecimen
- **Type status:** Other material. **Occurrence:** recordedBy: H.W. Byun, D.S. Choi,; individualCount: 2; sex: female; lifeStage: adult; occurrenceID: 1B07213C-4111-515A-AD74-3FD87D8DF645; **Taxon:** scientificName: Philheliuscoreanus; **Location:** country: South Korea; stateProvince: Gangwon-do; locality: Yonsei Univ. Mirae Campus, Maeji-ri, Heungeop-myeon, Wonju-si; verbatimLocality: 1998-07-14; verbatimCoordinates: 37°17'10"N 127°54'01"E; **Identification:** identifiedBy: Chan-Ouk Kim; dateIdentified: 2024; **Event:** eventDate: 1998-07-14; **Record Level:** language: en; institutionID: YSUW; collectionCode: Insects; basisOfRecord: PreservedSpecimen
- **Type status:** Other material. **Occurrence:** recordedBy: S.W. Suk; individualCount: 1; sex: male; lifeStage: adult; occurrenceID: 0EE6D1B4-8197-5DCE-9602-F587857E447B; **Taxon:** scientificName: Philheliuscoreanus; **Location:** country: South Korea; stateProvince: Gangwon-do; locality: Yonsei Univ. Mirae Campus, Maeji-ri, Heungeop-myeon, Wonju-si; verbatimLocality: 2008-07-07; **Identification:** identifiedBy: Chan-Ouk Kim; dateIdentified: 2024; **Event:** eventDate: 2008-07-07; **Record Level:** language: en; institutionID: YSUW; collectionCode: Insects; basisOfRecord: PreservedSpecimen
- **Type status:** Other material. **Occurrence:** recordedBy: S.W. Suk; individualCount: 1; sex: female; lifeStage: adult; occurrenceID: F3576A96-5AEF-5608-B199-BEBB3EE408BC; **Taxon:** scientificName: Philheliuscoreanus; **Location:** country: South Korea; stateProvince: Gangwon-do; locality: Yonsei Univ. Mirae Campus, Maeji-ri, Heungeop-myeon, Wonju-si; verbatimLocality: 2008-08-06; **Identification:** identifiedBy: Chan-Ouk Kim; dateIdentified: 2024; **Event:** eventDate: 2008-08-06; **Record Level:** language: en; institutionID: YSUW; collectionCode: Insects; basisOfRecord: PreservedSpecimen
- **Type status:** Other material. **Occurrence:** recordedBy: S.W. Suk; individualCount: 1; sex: female; lifeStage: adult; occurrenceID: FAA83FC1-12DD-533C-95FD-18D4819C11D6; **Taxon:** scientificName: Philheliuscoreanus; **Location:** country: South Korea; stateProvince: Gangwon-do; locality: Yonsei Univ. Mirae Campus, Maeji-ri, Heungeop-myeon, Wonju-si; verbatimLocality: 2009-06-16; **Identification:** identifiedBy: Chan-Ouk Kim; dateIdentified: 2024; **Event:** eventDate: 2009-06-16; **Record Level:** language: en; institutionID: YSUW; collectionCode: Insects; basisOfRecord: PreservedSpecimen
- **Type status:** Other material. **Occurrence:** recordedBy: S.S. Euo, S.H. Jeong,; individualCount: 1; sex: female; lifeStage: adult; occurrenceID: 870D2B9F-CE52-5DFE-9E99-4363EFF98D06; **Taxon:** scientificName: Philheliuscoreanus; **Location:** country: South Korea; stateProvince: Gangwon-do; locality: Yonsei Univ. Mirae Campus, Maeji-ri, Heungeop-myeon, Wonju-si; verbatimLocality: 2016-08-02; **Identification:** identifiedBy: Chan-Ouk Kim; dateIdentified: 2024; **Event:** eventDate: 2016-08-02; **Record Level:** language: en; institutionID: YSUW; collectionCode: Insects; basisOfRecord: PreservedSpecimen
- **Type status:** Other material. **Occurrence:** recordedBy: J.H. Choi; individualCount: 1; sex: male; lifeStage: adult; occurrenceID: F0DC126D-965E-5259-8520-FDD3E9D8BCC0; **Taxon:** scientificName: Philheliuscoreanus; **Location:** country: South Korea; stateProvince: Gangwon-do; locality: Yonsei Univ. Mirae Campus, Maeji-ri, Heungeop-myeon, Wonju-si; verbatimLocality: 2018-06-12; **Identification:** identifiedBy: Chan-Ouk Kim; dateIdentified: 2024; **Event:** eventDate: 2018-06-12; **Record Level:** language: en; institutionID: YSUW; collectionCode: Insects; basisOfRecord: PreservedSpecimen
- **Type status:** Other material. **Occurrence:** recordedBy: S.S. Euo; individualCount: 1; sex: female; lifeStage: adult; occurrenceID: ECDC5DCB-AD62-55BE-829D-EC9F87665284; **Taxon:** scientificName: Philheliuscoreanus; **Location:** country: South Korea; stateProvince: Gangwon-do; locality: Yonsei Univ. Mirae Campus, Maeji-ri, Heungeop-myeon, Wonju-si; verbatimLocality: 2018-06-24; **Identification:** identifiedBy: Chan-Ouk Kim; dateIdentified: 2024; **Event:** eventDate: 2018-06-24; **Record Level:** language: en; institutionID: YSUW; collectionCode: Insects; basisOfRecord: PreservedSpecimen
- **Type status:** Other material. **Occurrence:** recordedBy: D.S. Choi, S.K. Kim,; individualCount: 1; sex: female; lifeStage: adult; occurrenceID: 7E39C808-C51C-5866-B125-382FF7619CA5; **Taxon:** scientificName: Philheliuscoreanus; **Location:** country: South Korea; stateProvince: Gangwon-do; locality: Yongsu-gol, Seogok-ri, Panbu-myeon, Wonju-si; verbatimLocality: 1998-07-05; **Identification:** identifiedBy: Chan-Ouk Kim; dateIdentified: 2024; **Event:** eventDate: 1998-07-05; **Record Level:** language: en; institutionID: YSUW; collectionCode: Insects; basisOfRecord: PreservedSpecimen
- **Type status:** Other material. **Occurrence:** recordedBy: D.S. Choi, S.K. Kim,; individualCount: 1; sex: male; lifeStage: adult; occurrenceID: B862F0BF-3E23-5966-83D5-6475C07753EB; **Taxon:** scientificName: Philheliuscoreanus; **Location:** country: South Korea; stateProvince: Gangwon-do; locality: Mt. Baegunsan, Seogok-ri, Panbu-myeon, Wonju-si; verbatimLocality: 1998-07-17; verbatimCoordinates: 37°14'59"N 127°57'46"E; **Identification:** identifiedBy: Chan-Ouk Kim; dateIdentified: 2024; **Event:** eventDate: 1998-07-17; **Record Level:** language: en; institutionID: YSUW; collectionCode: Insects; basisOfRecord: PreservedSpecimen
- **Type status:** Other material. **Occurrence:** recordedBy: S.S. Euo, C.O. Kim, J.H. Choi; individualCount: 1; sex: male; lifeStage: adult; occurrenceID: EA9C5AD4-C014-5A7B-A917-751951A0563D; **Taxon:** scientificName: Philheliuscoreanus; **Location:** country: South Korea; stateProvince: Gangwon-do; locality: Mt. Taehwasan, Heungwol-ri, Yeongwol-eup, Yeongwol-gun; verbatimLocality: 2018-08-01; verbatimCoordinates: 37°07'03.3"N 128°29'07.4"E; **Identification:** identifiedBy: Chan-Ouk Kim; dateIdentified: 2024; **Event:** eventDate: 2018-08-01; **Record Level:** language: en; institutionID: YSUW; collectionCode: Insects; basisOfRecord: PreservedSpecimen
- **Type status:** Other material. **Occurrence:** recordedBy: S.S. Euo, C.O. Kim, J.H. Choi; individualCount: 5; sex: female; lifeStage: adult; occurrenceID: 35C8C0D2-7C2E-5CD6-8304-D0A36A410008; **Taxon:** scientificName: Philheliuscoreanus; **Location:** country: South Korea; stateProvince: Gangwon-do; locality: Mt. Taehwasan, Heungwol-ri, Yeongwol-eup, Yeongwol-gun; verbatimLocality: 2018-08-01; verbatimCoordinates: 37°07'03.3"N 128°29'07.4"E; **Identification:** identifiedBy: Chan-Ouk Kim; dateIdentified: 2024; **Event:** eventDate: 2018-08-01; **Record Level:** language: en; institutionID: YSUW; collectionCode: Insects; basisOfRecord: PreservedSpecimen
- **Type status:** Other material. **Occurrence:** recordedBy: S.S. Euo, C.O. Kim, J.H. Choi; individualCount: 1; sex: male; lifeStage: adult; occurrenceID: DC172158-4A4E-5111-BA0A-64BC3D5A9F1F; **Taxon:** scientificName: Philheliuscoreanus; **Location:** country: South Korea; stateProvince: Gangwon-do; locality: Mt. Taehwasan, Heungwol-ri, Yeongwol-eup, Yeongwol-gun; verbatimLocality: 2019-07-30; verbatimCoordinates: 37°07'03.3"N 128°29'07.4"E; **Identification:** identifiedBy: Chan-Ouk Kim; dateIdentified: 2024; **Event:** eventDate: 2019-07-30; **Record Level:** language: en; institutionID: YSUW; collectionCode: Insects; basisOfRecord: PreservedSpecimen
- **Type status:** Other material. **Occurrence:** recordedBy: S.S. Euo, C.O. Kim, J.H. Choi; individualCount: 7; sex: female; lifeStage: adult; occurrenceID: 5D950140-760D-5DAC-BB0E-200E31D33D02; **Taxon:** scientificName: Philheliuscoreanus; **Location:** country: South Korea; stateProvince: Gangwon-do; locality: Mt. Taehwasan, Heungwol-ri, Yeongwol-eup, Yeongwol-gun; verbatimLocality: 2019-07-30; verbatimCoordinates: 37°07'03.3"N 128°29'07.4"E; **Identification:** identifiedBy: Chan-Ouk Kim; dateIdentified: 2024; **Event:** eventDate: 2019-07-30; **Record Level:** language: en; institutionID: YSUW; collectionCode: Insects; basisOfRecord: PreservedSpecimen
- **Type status:** Other material. **Occurrence:** recordedBy: S.S. Euo, C.O. Kim, J.H. Choi; individualCount: 2; sex: male; lifeStage: adult; occurrenceID: EA0B863C-250C-5634-8634-B191E9754168; **Taxon:** scientificName: Philheliuscoreanus; **Location:** country: South Korea; stateProvince: Gangwon-do; locality: Mt. Taehwasan, Heungwol-ri, Yeongwol-eup, Yeongwol-gun; verbatimLocality: 2020-07-02; verbatimCoordinates: 37°07'03.3"N 128°29'07.4"E; **Identification:** identifiedBy: Chan-Ouk Kim; dateIdentified: 2024; **Event:** eventDate: 2020-07-02; **Record Level:** language: en; institutionID: YSUW; collectionCode: Insects; basisOfRecord: PreservedSpecimen
- **Type status:** Other material. **Occurrence:** recordedBy: S.S. Euo, C.O. Kim, J.H. Choi; individualCount: 6; sex: female; lifeStage: adult; occurrenceID: 07F10864-9395-5976-9CCF-85568C2850B7; **Taxon:** scientificName: Philheliuscoreanus; **Location:** country: South Korea; stateProvince: Gangwon-do; locality: Mt. Taehwasan, Heungwol-ri, Yeongwol-eup, Yeongwol-gun; verbatimLocality: 2020-07-02; verbatimCoordinates: 37°07'03.3"N 128°29'07.4"E; **Identification:** identifiedBy: Chan-Ouk Kim; dateIdentified: 2024; **Event:** eventDate: 2020-07-02; **Record Level:** language: en; institutionID: YSUW; collectionCode: Insects; basisOfRecord: PreservedSpecimen
- **Type status:** Other material. **Occurrence:** recordedBy: H.C. Park; individualCount: 1; sex: female; lifeStage: adult; occurrenceID: C17157E2-F65B-52CA-8745-79F3A0B1A856; **Taxon:** scientificName: Philheliuscoreanus; **Location:** country: South Korea; stateProvince: Gangwon-do; locality: Gajeong-ri, Nam-myeon, Chuncheon-si,; verbatimLocality: 2013-06-21; **Identification:** identifiedBy: Chan-Ouk Kim; dateIdentified: 2024; **Event:** eventDate: 2013-06-21; **Record Level:** language: en; institutionID: NASIC; collectionCode: Insects; basisOfRecord: PreservedSpecimen

#### Re-description

**Figure 2.**
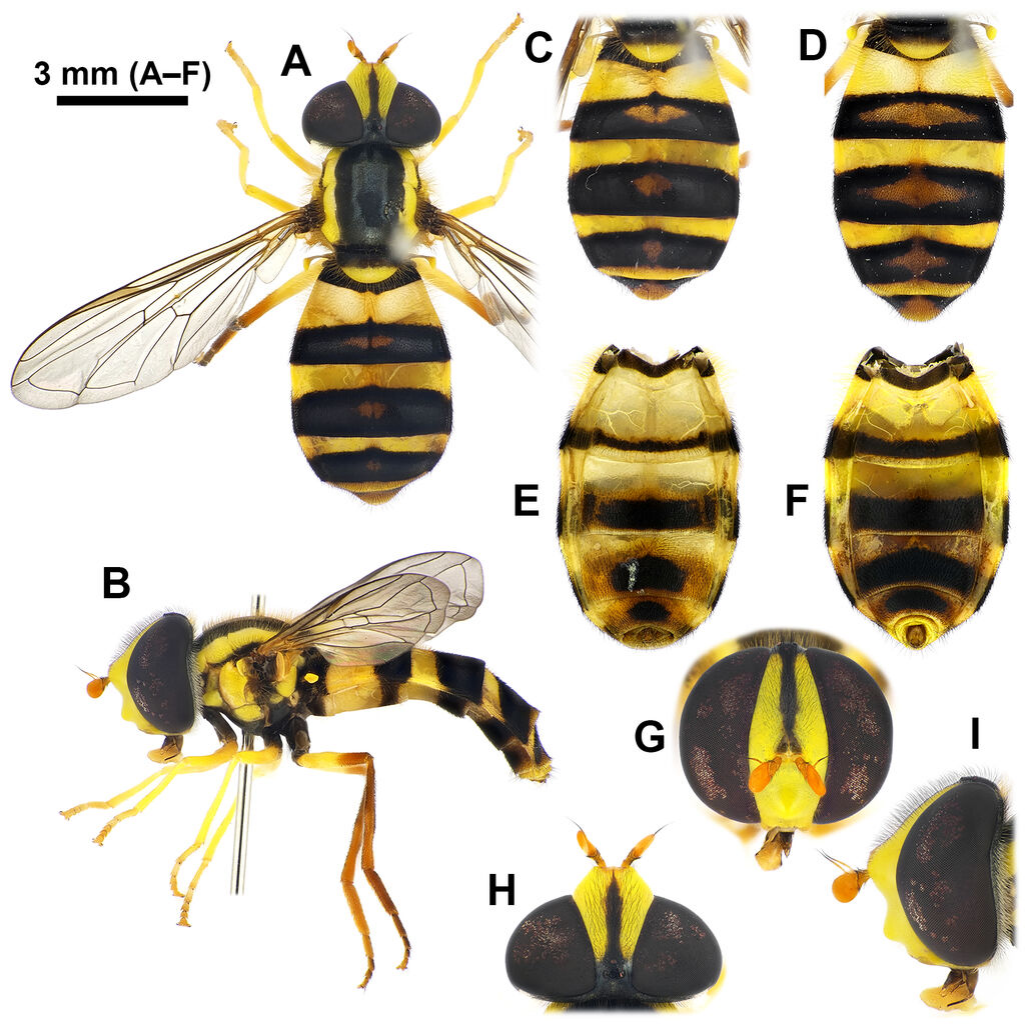
*Philheliuscoreanus*, females. **A, B** body, dorsal and lateral view; **C–F** abdomen: dorsal view (**C, D**); ventral view (**E, F**); **G–I** head: frontal view (**G**); dorsal view (**H**); lateral view (**I**).

**Figure 3.**
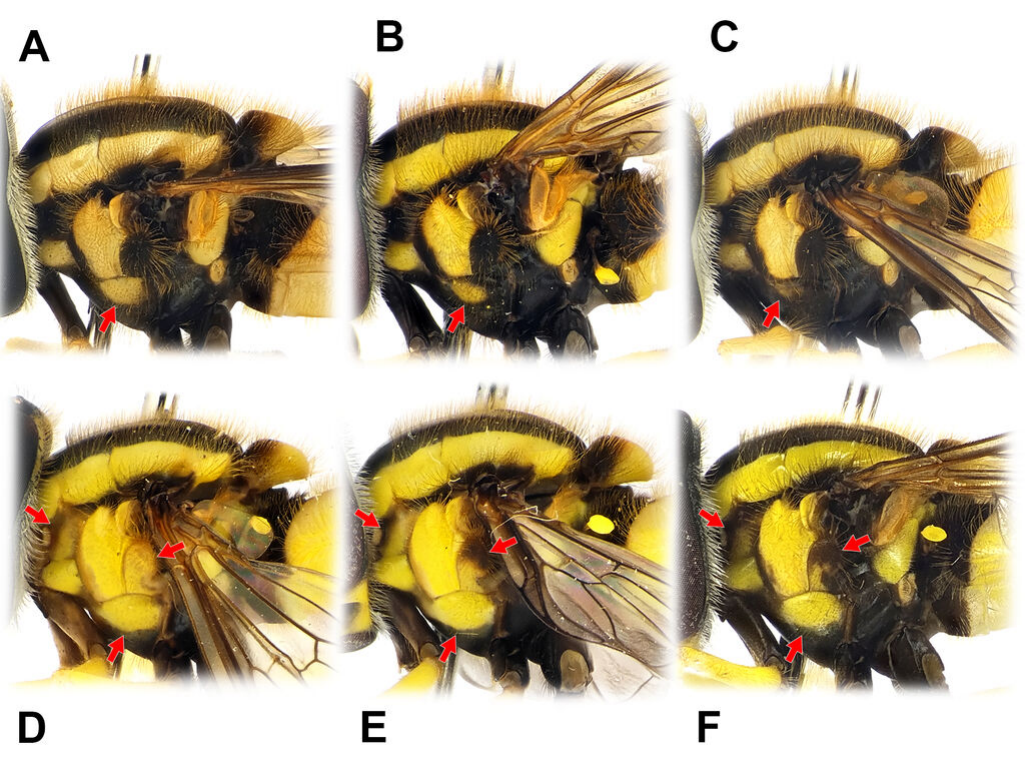
Thorax of *Philheliuscoreanus* in lateral view, showing variation. **A–C** males; **D–F** females. Red arrows indicate areas with high variation.

**Figure 4.**
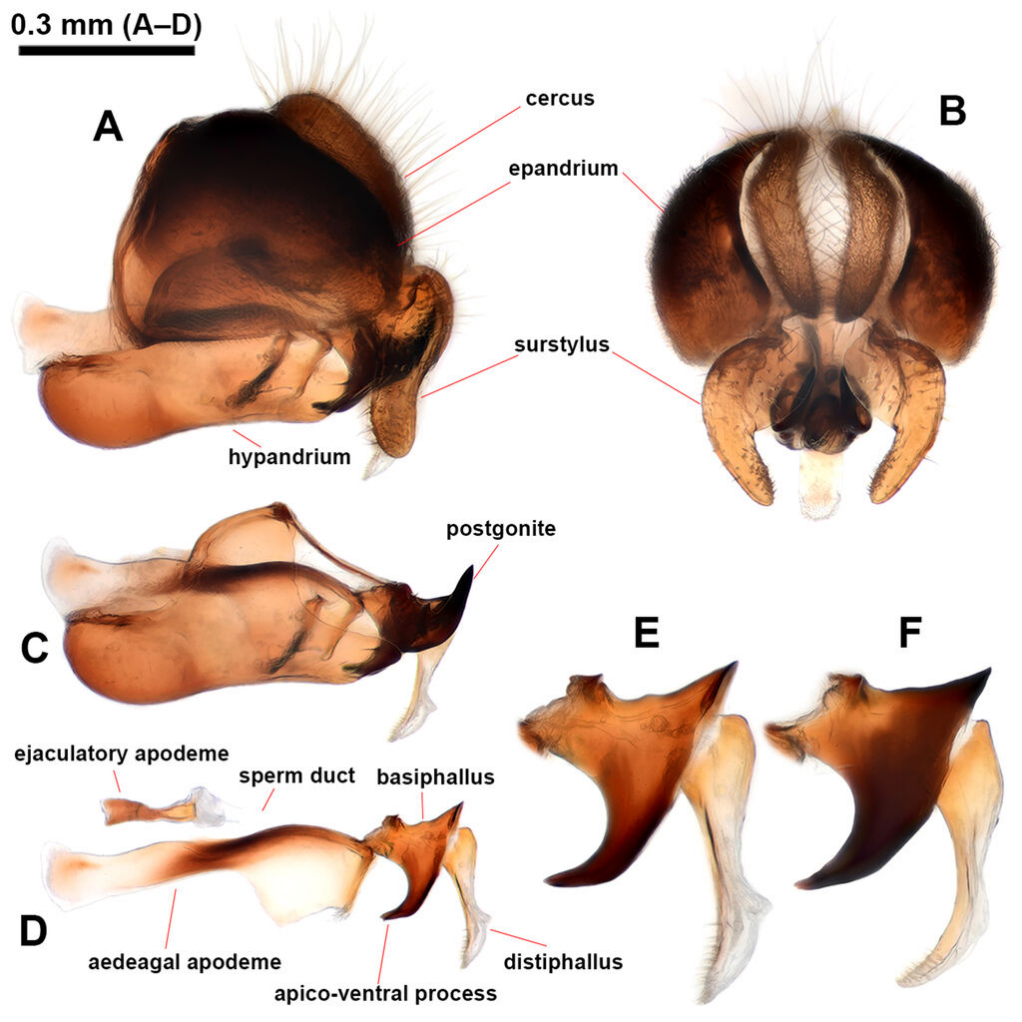
Male genitalia of *Philheliuscoreanus*. **A** lateral view; **B** caudal view; **C** hypandrium, lateral view; **D** phallus, lateral view; **E, F** basiphallus and distiphallus, lateral view, showing the general range of variation.

**Figure 5.**
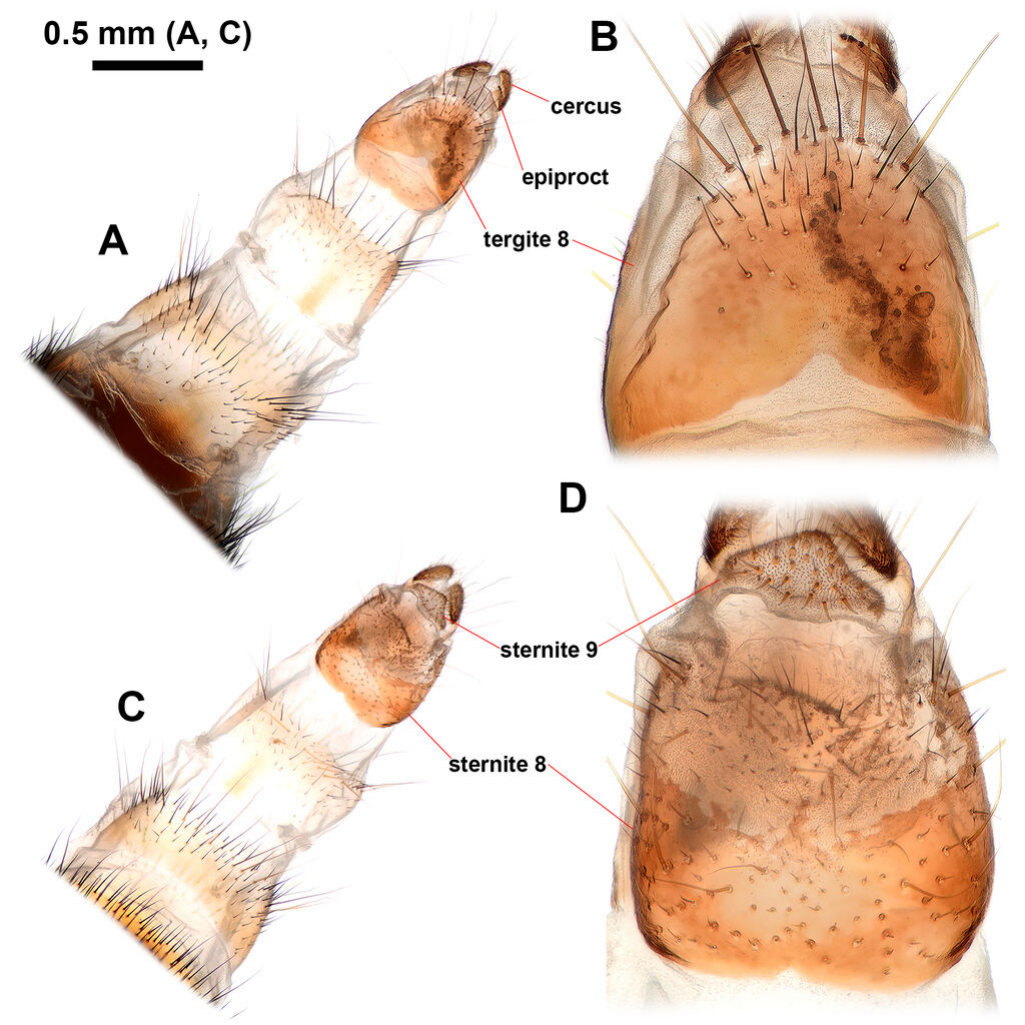
Female terminalia of *Philheliuscoreanus*. **A, B** dorsal view; **C, D** ventral view.

**Male** (Fig. 1, Fig. 3A–C and Fig. 4). Lengths and ratios: body length 8.7–13.5 mm; wing length 6.8–9.9 mm; head ratio 0.68–0.76; face ratio 0.28–0.40; eye ratio 0.45–0.52; gena-eye ratio (genal height extremely narrow - not measured); antenna-head ratio 0.41–0.45; postpedicel-pedicel ratio 1.83–2.39; arista-antenna ratio 0.85–1.05; wing ratio 3.47–3.62; wing-thorax ratio 2.14–2.69; vein M ratio 1.93–2.97; vein R_4+5_ ratio 3.02–3.48. **Head** (Fig. 1G–I): holoptic with eye contiguity as long as vertical triangle; compound eye dark brown with slight purplish tinge, bare; vertex black postero-marginally with yellow pruinosity, anteriorly with wavy black hairs, postero-marginally with wavy brownish-yellow hairs; frons largely yellow, but medially slightly darkened, with black hairs; lunule largely brownish-yellow, bare; antenna entirely brownish-yellow to pale brown; face yellow ground colour with brownish-yellow and black hairs; facial knob rounded, almost bare; gena largely yellow, but partially brownish-black, with brownish-yellow hairs. **Thorax** (Fig. 1A and Fig. 3A–C): largely black, partially with yellow maculae and with slightly wavy brownish-yellow hairs; scutum with slightly subshiny greyish pruinosity, medial scutal area with pair of longitudinal greyish pruinose stripe (can be observed with appropriate lighting) interrupted at anterior 2/3; scutum with distinct yellow latero-marginal stripes; scutellum about anterior 1/2–2/3 black and about posterior 1/3–1/2 yellow, with black and brownish-yellow hairs; pleura largely black with yellow spots on proepimeron, about posterior 3/4 of posterior anepisternum, upper part of katepisternum and katatergite, of these, katepisternal spot shows various intensities (Fig. 3A–C show a range of variation); anterior anepisternum, about anterior 1/3 of posterior anepisternum, dorsomedial anepimeron, posterior anepimeron, meron, anatergite, mediotergite and metasternum bare; katepisternum with separated upper and lower wavy brownish-yellow hair patches; halter with stem brown to yellow, knob yellow (sometimes entirely brown to dark brown). **Legs** (Fig. 1B): coxae and trochanters dark brown to black, with black hairs; fore- and mid-legs largely yellow with brownish tarsi, with brownish-yellow hairs; hind leg largely pale brown to brown, except for yellow basal 1/2 of hind femur, with brownish-yellow and black hairs. **Wing** (Fig. 1A): largely hyaline with slight brownish tinge; veins brownish-yellow to brownish-black; pterostigma pale brown; wing membrane largely covered with microtrichiae, except for basal areas; upper and lower calypters pale yellow to yellow with long brownish-yellow marginal hairs. **Abdomen**: Abdominal tergites margined, black ground colour with yellow markings, usually with brownish-yellow subposterior area on tergites 2–4 in various intensities (Fig. 1A–D show rough range of variation), with wavy brownish-yellow and black hairs; tergite 1 black; tergite 2 with pair of rounded yellow lateral spots reaching lateral margins; tergite 3 with yellow antero-marginal transverse band postero-medially slightly incised; tergite 4 with yellow antero- and postero-marginal transverse bands, antero-marginal band postero-medially slightly incised; tergite 5 antero-lateral margins yellow, medially brownish-black and the rest of the posterior area brownish-yellow; abdominal sternites largely pale yellow to yellow, gradually slightly darkened towards the rear, with brownish-black markings in various intensities (Fig. 1E and F show rough range of variation), with brownish-yellow and black hairs; sternite 1 with antero-marginal brownish-black transverse band; sternite 2 with narrow brownish-black subposterior transverse band antero-medially slightly extended and less than 1/6 length of sternite 2; sternites 3–4 subposteriorly with transversally wide brownish-black medial spot and postero-lateral margin brownish-black; sternites 5–8 brownish-yellow to pale brown with brownish-black spot on sternite 8, largely with short black hairs. **Male genitalia** (Fig. 4): epandrium slightly longer than height in lateral view (Fig. 4A); surstylus comma-shaped in caudal view (basal width about 3x apical width when orientated to show broadest area) (Fig. 4B), basally with long brownish-yellow hairs, apically with short hairs (Fig. 4A and B); hypandrium without lingula (Fig. 4C); postgonite upward sickle shape in lateral view (Fig. 4C); distiphallus narrow and dorsal side slightly concave at the middle in lateral view (Fig. 4D–F); basiphallus with short apico-dorsal and long apico-ventral processes, apico-ventral process distinctly curved posteriorly (Fig. 4D–F show rough range of variation). **Female** (Fig. 2, Fig. 3D–F and Fig. 5). Similar to males, except for the following characteristics. **Head** (Fig. 2G–I): dichoptic eyes, with vertex about 0.17x as wide as head in dorsal view; frons largely yellow with brownish-black longitudinal stripe from brownish-black posterior margin to above area of the lunule, largely with black hairs, but antero-laterally with brownish-yellow hairs. **Thorax** (Fig. 2A and Fig. 3D–F): yellow spots on pleura variable and covering larger areas than in males (Fig. 3D–F vs. A–C also show a range of variation). **Abdomen**: abdominal tergite 2 with yellow transverse band (Fig. 2A–D also show rough range of variation); sternites 2–4 with brownish-black subposterior band or spot larger than in males (Fig. 2E, and F show rough range of variation); sternite 5 with brownish-black medial spot of various sizes. **Female terminalia** (Fig. 5): tergites 6 and 7 and sternites 6 and 7 with short black hairs on apical half, with relatively long apico-marginal hairs (Fig. 5A and C); tergite 8 sparsely covered with short black hairs on posterior 1/2–3/4, apico-marginally with relatively long brownish hairs and longitudinally and narrowly microtrichose; tergite 8 with a large and pointed-arch-shaped brownish sclerite antero-medially slightly incised and the rest of the postero-marginal area membraneous (Fig. 5B); epiproct with a pair of sclerotised plates; cercus short, brown, with brownish hairs; sternite 8 sparsely with short brown hairs, anteriorly with latero-marginally peaked brownish sclerite (antero-medially slightly peaked) and the rest of the posterior area membraneous; sternite 9 triangular with short and stout brownish hairs (Fig. 5D).

#### Distribution

Republic of Korea, China, southern Russian Far East (Barkalov and Mutin 2018, Huo 2020).

#### Remarks

*Philheliuscoreanus* was originally described, based on Korean male specimens (Shiraki 1930) and our analysis of Korean males in this study was consistent with this description. This species can be distinguished from congeners by the following combination of characteristics: (1) compound eye almost bare, but sparsely covered with short yellow hairs; (2) scutellum entirely covered with brownish-yellow hairs, with a few black hairs mixed (Fig. 3); (3) yellow transverse bands on tergites 3 and 4 are not interrupted (Fig. 1A, C, D, Fig. 2A, C and D); and (4) black transverse band on sternite 2 less than 1/6 the length of the sternite (Fig. 1E, F, Fig. 2E and F). Although it closely resembles *P.anisomorphum* (Huo, Ren & Zheng, 2007), *P.coreanus* can be recognised by eyes nearly bare (Fig. 1I and Fig. 2I), posterior part of vertex covered with brownish-yellow hairs and wing basally with bare areas.

Females were first described by Violovitsh (1975), based on Russian specimens collected in Primorsky Krai, although intraspecific variation was not accurately represented. Our Korean female specimens were consistent with the description of Russian females, except for a difference in pleural spots. Violovitsh (1975) described three yellow spots on the notopleuron, anepisternum and katepisternum; however, all *Philhelius* species have a pair of yellow latero-marginal stripes extending from the postpronotal lobe through the notopleuron to the postalar callus. The notopleuron is always yellow because it is contained in a yellow stripe, making the identification of a yellow spot unlikely. Therefore, we assume that the yellow spot on the notopleuron is a descriptive mistake (e.g. the notopleuron was confused with another pleural part). No females with exactly three yellow spots on the pleura were identified; instead, various variants of yellow spots on the anterior anepisternum, posterior anepisternum, proepimeron, katepisternum and anepimeron were observed. To a lesser extent than in females, variation is also observed in males (Fig. 3A–C vs. D–F), suggesting that the yellow spot on the pleura of *P.coreanus* is a highly variable character.

Additionally, Violovitsh’s (1975) description was based on only three specimens, which is a very small sample size and leaves open the possibility that an extreme phenotype (somewhat more pronounced than in Fig. 3F) was collected by chance. Alternatively, the specimens in question might represent a different species altogether or the discrepancy could be attributable to a descriptive error. Therefore, future studies should re-examine the original material of *P.coreanus* described by Violovitsh (1975), ideally incorporating both morphological and molecular analyses, to clarify its identity.

## Analysis

### Complete mitochondrial genome of Philheliuscoreanus

**Figure 6.**
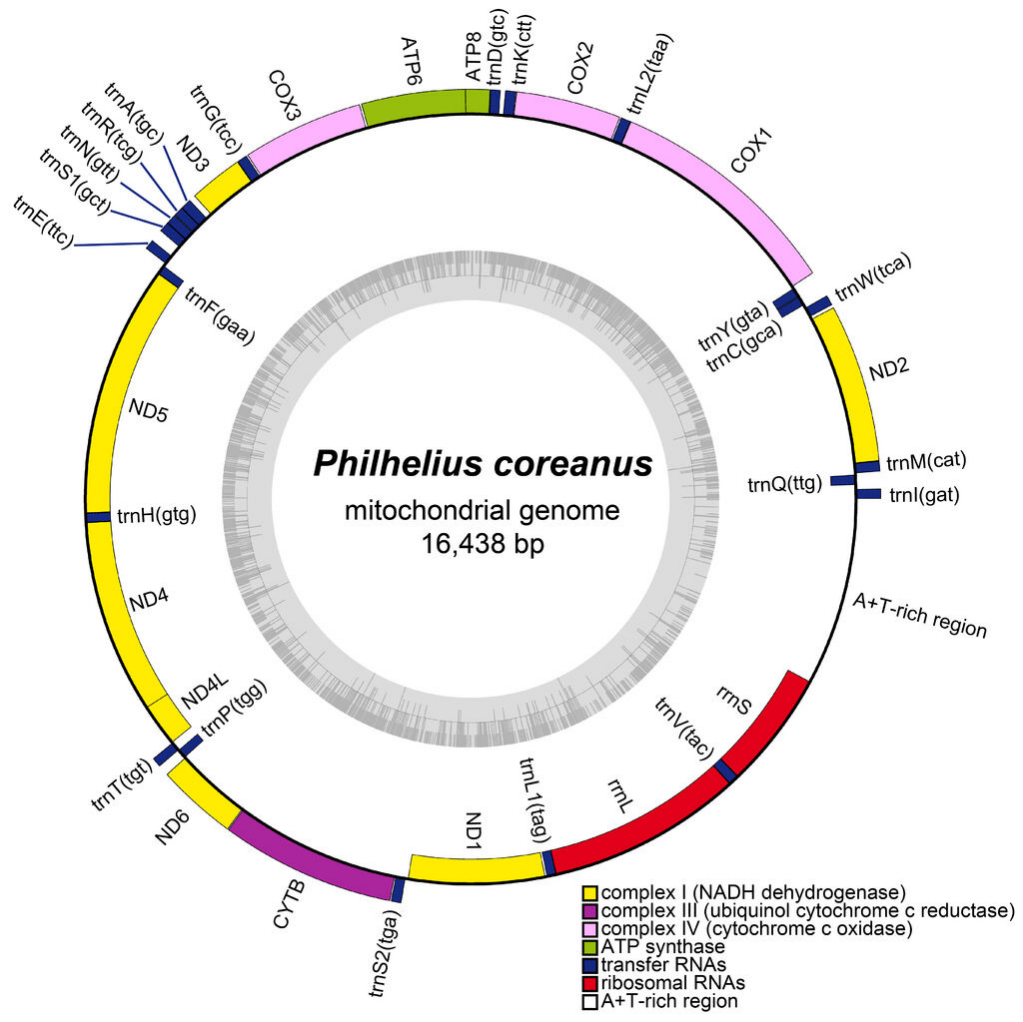
Circular mitogenome maps for *Philheliuscoreanus*. Colour codes of different types of genes are shown in the key. Genes encoded on the forward strand are drawn on the outside of the circle and those on the reverse strand are drawn inside of the circle. The inner circle (grey) shows the GC content.

**Figure 7.**
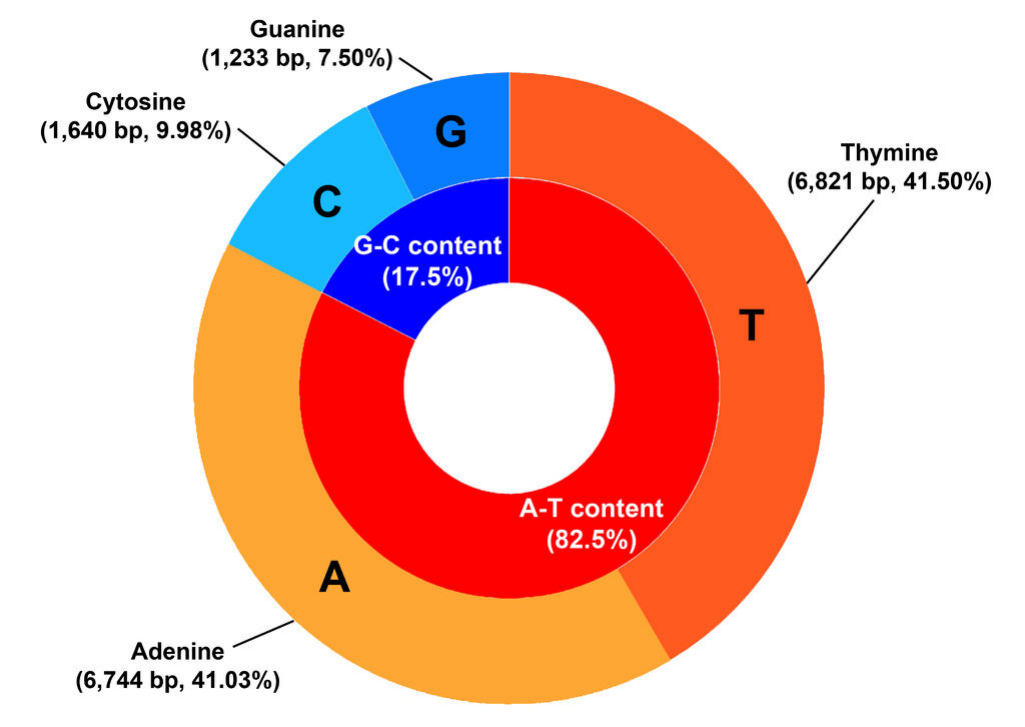
Overall nucleotide base composition of *Philheliuscoreanus*. A-T and G-C contents, with the frequencies of each base.

The mitogenome of *P.coreanus* had 16,438 bp (GenBank accession number: PQ218350) and included 37 genes (Fig. 6). The nucleotide composition of the whole *P.coreanus* mitogenome was 41% adenine, 41.5% thymine, 10% cytosine and 7.5% guanine, with a GC content of 17.5%. The AT-skew (-0.005676) and GC-skew (-0.1417) were negative (Fig. 7). Information about the 37 genes (two rRNA genes, 13 PCGs, 22 tRNA genes) and an A+T-rich region in the mitogenome of *P.coreanus* is given in Table 2.

**Figure 8.**
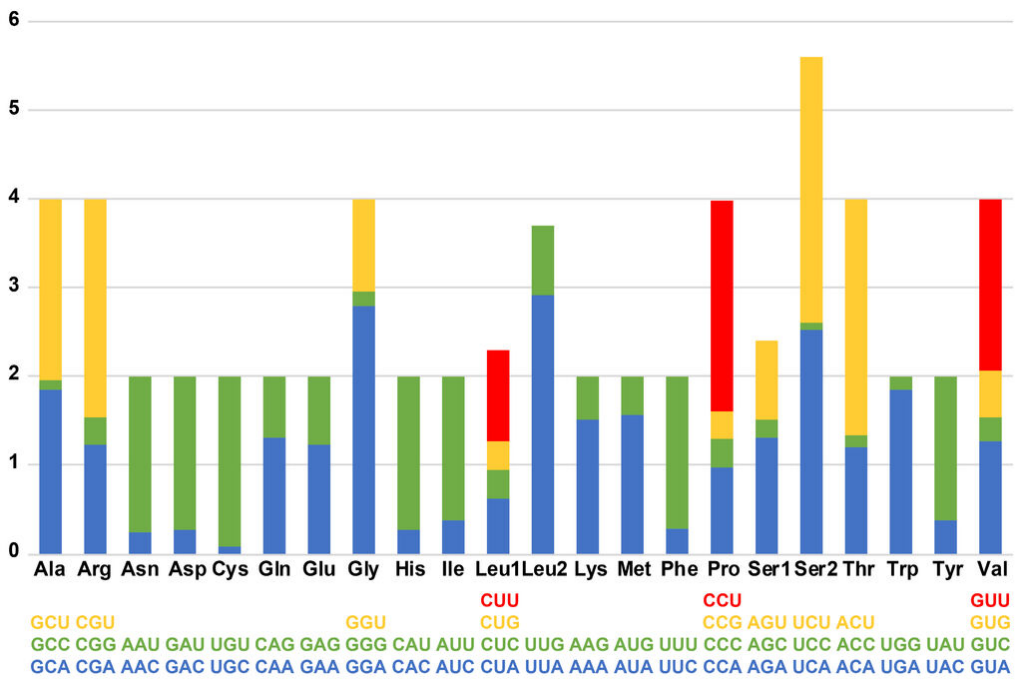
Relative synonymous codon usage (RSCU) in protein-coding genes in the *Philheliuscoreanus* mitogenome.

Of the 13 PCGs, 11 were initiated by ATN codons and used a typical TAA termination codon. *COX1* was initiated by TTG and terminated with a single T, completed by additional A residues. *ND5* was initiated by the ATG start codon and terminated by TA, with an additional A residue. Amino-acid usage and relative synonymous codon usage (RSCU) in PCGs of *P.coreanus* are given in Fig. 8 and Table 3. There were 3,378 total codons in the 13 PCGs of *P.coreanus*. The five most frequent codons were as follows (accounting for 42.9% of the total): AUA (Met) (6.63%), AUU (Ile) (7.90%), UAU (Tyr) (8.64%), UUU (Phe) (9.65%) and UUA (Leu) (10.1%) (Table 3).

**Figure 9.**
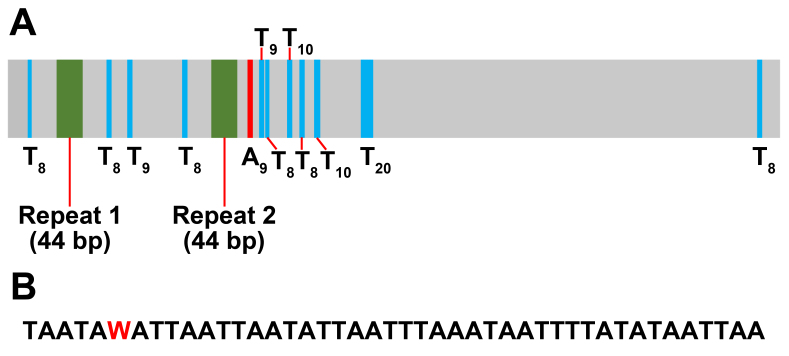
Schematic map showing the 1,293 bp long A+T-rich region of *Philheliuscoreanus* mitochondrial genome. **A** Structure of the A+T-rich region. Poly-A and -T nucleotides are presented as A and T, with a subscript indicating the number of repetitions; **B** Consensus alignment of the two repeat regions (Repeats 1 and 2). Nucleotides written in red indicate sequence variation between the two repeats.

The *P.coreanus* mitogenome contained 22 typical tRNAs, ranging in length from 65 bp (*trnH*) to 72 bp (*trnA* and *trnV*) (Table 2). The total length of tRNAs was 1,480 bp, accounting for approximately 9.0035% of the complete mitogenome. Eight tRNAs were encoded by the N-strand and the remaining 14 were encoded by the J-strand. Two rRNA genes, *rrnS* and *rrnL*, were identified; rrnS was 841 bp and was located between *trnV* and the A+T-rich region and *rrnL* was 1,346 bp long and was located between *trnL1* and *trnV*. The A+T-rich region was located between the *rrnS* and *trnI* genes and was 1,293 bp long. The A+T-rich regions of Syrphini genomes included in our analysis were 873–1,491 bp, with an average length of 1228.3 bp, similar to the length observed in *P.coreanus*. The A+T-rich region was composed of adenine 43.4% (561 bp) and thymine 51.4% (664 bp), which accounted for approximately 94.7% of the whole region. In addition, two repeat regions (repeats 1 and 2; Fig. 9A) were detected at positions 82–125 bp (repeat 1) and 340–383 bp (repeat 2). These repeat regions were both 44 bp long, showed 97.7% sequence homology and consisted of only adenine and thymine bases (Fig. 9B). They differed only at the 6^th^ nucleotide, which was adenine in repeat 1 and thymine in repeat 2. Within the non-repeat sequences, there were poly-A and -T sites consisting of consecutive adenine or thymine (Fig. 9A). A single poly-A site consisting of nine adenine bases (A_9_) was detected at position 401–409 bp. With respect to poly-T sites, there were eight thymine bases (T_8_) at positions 33–40, 166–173, 293–300, 431–438, 489–496 and 1,256–1,263 bp, nine thymine bases (T_9_) at positions 200–208 and 421–429, ten thymine bases (T_10_) at positions 467–476 and 513–522 bp and 20 thymine bases (T_20_) at position 592–611 bp.

### Phylogenetic Analysis

The concatenated alignment of 13 PCGs of 25 syrphid species, including *Philheliuscoreanus*, had 11,305 bp, although five species had missing PCGs. We conducted a phylogenetic analysis, based on the concatenated alignment using the ML method.

**Figure 10.**
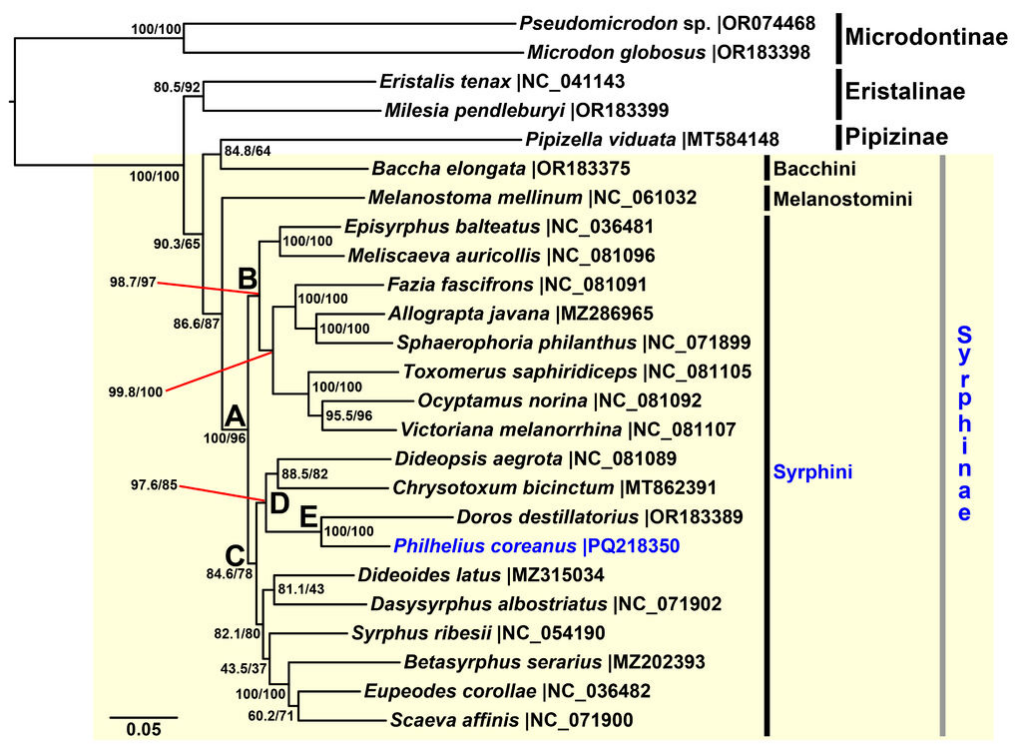
Maximum-Likelihood phylogenetic tree, based on 13 concatenated protein-coding genes in 24 mitogenomes retrieved from GenBank (https://www.ncbi.nlm.nih.gov/genbank/; as of Sept 2024) and the newly-obtained mitogenome of Korean *Philheliuscoreanus*. Numbers at nodes indicate the SH-aLRT (left) and ultrafast bootstrap values (after 1000 replications; right). Subfamily and tribe names are labelled with vertical bars to the right of the tree; black bars indicate a higher taxon for which only a single species was included in the analysis or a higher taxon resolved as monophyletic; grey bar indicates a higher taxon that is not resolved as monophyletic. The Syrphinae are indicated with a yellow shaded box. A–E indicate clades discussed in the text. Scale bar indicates 0.05 substitutions per site.

In the ML tree, the monophyly of the Syrphinae was not recovered: the tribe Syrphini clustered with Melanostomini and Bacchini grouped with Pipizinae (Fig. 10). Instead, the monophyly of Syrphini was strongly supported (Fig. 10A), with two major lineages identified within the tribe (Fig. 10B and C).

*Philheliuscoreanus* was shown to be closely related to *Dorosdestillatorius* Mik, 1885, with maximum support (Fig. 10E) suggesting that *Philhelius* and *Doros* are sister genera. Furthermore, our ML tree showed that the *Philhelius* + *Doros* clade is a sister group to the *Dideopsis* + *Chrysotoxum* clade, with a relatively high support value (Fig. 10D).

## Discussion

The phylogenetic analysis showed that *Philheliuscoreanus* is closely related to *Dorosdestillatorius*, forming a sister group with maximum support (Fig. 10E). This relationship is consistent with the morphological similarities between the two genera (Dušek and Láska 1967, Shatalkin 1975, Rotheray and Gilbert 1999) and previous molecular phylogenetic studies (Mengual et al. 2018, Mengual et al. 2023).

Mengual et al. (2023) found that the *Philhelius* + *Doros* clade is sister to *Dideoides*. On the other hand, Wong et al. (2023) placed *Doros* as a sister group to the *Dideopsis* + *Chrysotoxum* clade, though their analysis excluded both *Philhelius* and *Dideoides*. Our ML tree supports the placement of the *Philhelius* + *Doros* clade as a sister group to the *Dideopsis* + *Chrysotoxum* clade, with relatively high support (Fig. 10D), consistent with the findings for *Doros* in Wong et al. (2023).

Furthermore, our analysis supports the monophyly of Syrphini (Fig. 10A) and identifies two major lineages within this tribe (Fig. 10B and C), consistent with recent studies (Mengual et al. 2008, Wong et al. 2023, Mengual et al. 2023). However, the monophyly of Syrphinae was not recovered in our analysis, likely due to the limited taxon sampling. Specifically, the grouping of *Pipizellaviduata* and *Bacchaelongata* was supported by relatively low statistical values, possibly influenced by the inclusion of only two PCGs from *P.viduata*, which is significantly fewer than the full complement of 13 PCGs used for other taxa.

While the sister-group relationship between *Philhelius* and *Doros* was strongly supported, the exact placement of the *Philhelius + Doros* clade within Syrphini remains unclear. Therefore, future studies should incorporate and analyse additional mitochondrial genomes from taxa not included in the present analysis (e.g. *Epistrophe*, *Leucozona* Schiner, 1860, *Melangyna* Verrall, 1901 and *Paragus* Latreille, 1804) to clarify these relationships.

## Supplementary Material

XML Treatment for Philhelius

XML Treatment for Philhelius coreanus

## Figures and Tables

**Table 1.** Information on the species included in the molecular analysis.

**Subfamily**	**Tribe**	**Species**	**GenBank accession number**	**Number of PCGs used**	**References**
Eristalinae	Eristalini	*Eristalistenax*	NC_041143	13	Li et al. (2017)
	Milesiini	*Milesiapendleburyi*	OR183399	3	Wong et al. (2023)
Microdontinae	Microdontini	*Microdonglobosus*	OR183398	13	Wong et al. (2023)
	Microdontini	*Pseudomicrodon* sp.	OR074468	13	Wong et al. (2023)
Pipizinae	Pipizini	*Pipizellaviduata*	MT584148	2	DNAmark project (2020), unpublished
Syrphinae	Bacchini	*Bacchaelongata*	OR183375	2	Wong et al. (2023)
	Melanostomini	*Melanostomamellinum*	NC_061032	13	Liu et al. (2022)
	Syrphini	*Allograptajavana*	MZ286965	13	Li et al. (2023)
		*Betasyrphusserarius*	MZ202393	13	Li et al. (2023)
		*Chrysotoxumbicinctum*	MT862391	3	DNAmark project (2020), unpublished
		*Dasysyrphusalbostriatus*	NC_071902	13	NCBI Genome Project (2023), unpublished (Direct Submission)
		*Dideoideslatus*	MZ315034	13	Li et al. (2023)
		*Dideopsisaegrota*	NC_081089	13	Wong et al. (2023)
		*Dorosdestillatorius*	OR183389	8	Wong et al. (2023)
		*Episyrphusbalteatus*	NC_036481	13	Pu et al. (2017)
		*Eupeodescorollae*	NC_036482	13	Pu et al. (2017)
		*Faziafascifrons*	NC_081091	13	Wong et al. (2023)
		*Meliscaevaauricollis*	NC_081096	13	Wong et al. (2023)
		*Ocyptamusnorina*	NC_081092	13	Wong et al. (2023)
		*Philheliuscoreanus*	PQ218350	13	This study
		*Scaevaaffinis*	NC_071900	13	NCBI Genome Project (2023), unpublished (Direct Submission)
		*Sphaerophoriaphilanthus*	NC_071899	13	NCBI Genome Project (2023), unpublished (Direct Submission)
		*Syrphusribesii*	NC_054190	13	Chen et al. (2021)
		*Toxomerussaphiridiceps*	NC_081105	13	Wong et al. (2023)
		*Victorianamelanorrhina*	NC_081107	13	Wong et al. (2023)

**Table 2.** Features of protein-coding genes in the mitochondrial genome of *Philheliuscoreanus*.

**Gene**	**Direction**	**Start**	**End**	**Size (bp)**	**Anticodon**	**Start Codon**	**Stop Codon**
trnI	F	1	67	67	GAT	-	-
trnQ	R	97	165	69	TTG	-	-
trnM	F	176	243	68	CAT	-	-
ND2	F	244	1272	1029	-	ATT	TAA
trnW	F	1291	1359	69	TCA	-	-
trnC	R	1380	1447	68	GCA	-	-
trnY	R	1449	1514	66	GTA	-	-
COX1	F	1528	3061	1534	-	TTG	T
trnL2	F	3062	3127	66	TAA	-	-
COX2	F	3134	3817	684	-	ATG	TAA
trnK	F	3819	3889	71	CTT	-	-
trnD	F	3918	3983	66	GTC	-	-
ATP8	F	3984	4145	162	-	ATC	TAA
ATP6	F	4139	4816	678	-	ATG	TAA
COX3	F	4827	5615	789	-	ATG	TAA
trnG	F	5623	5689	67	TCC	-	-
ND3	F	5690	6043	354	-	ATT	TAA
trnA	F	6082	6153	72	TGC	-	-
trnR	F	6153	6216	64	TCG	-	-
trnN	F	6218	6284	67	GTT	-	-
trnS1	F	6285	6352	68	GCT	-	-
trnE	F	6433	6498	66	TTC	-	-
trnF	R	6518	6581	64	GAA	-	-
ND5	R	6582	8314	1733	-	ATG	TA
trnH	R	8315	8379	65	GTG	-	-
ND4	R	8380	9720	1341	-	ATG	TAA
ND4L	R	9714	10010	297	-	ATG	TAA
trnT	F	10013	10077	65	TGT	-	-
trnP	R	10078	10143	66	TGG	-	-
ND6	F	10146	10670	525	-	ATT	TAA
CYTB	F	10674	11810	1137	-	ATG	TAA
trnS2	F	11816	11883	68	TGA	-	-
ND1	R	11900	12838	939	-	ATA	TAA
trnL1	R	12849	12914	66	TAG	-	-
rrnL	R	12914	14259	1346	-	-	-
trnV	R	14260	14331	72	TAC	-	-
rrnS	R	14332	15145	841	-	-	-
A+T-rich region	F	15146	16438	1293	-	-	-

**Table 3.** Relative synonymous codon usage (RSCU) in protein-coding genes in the *Philheliuscoreanus* mitogenome.

**Amino acid**	**Codon**	**Count**	**RSCU**	**Amino acid**	**Codon**	**Count**	**RSCU**	**Amino acid**	**Codon**	**Count**	**RSCU**
Ala	GCA	18	1.85	His	CAC	13	0.26	Pro	CCU	22	2.38
Ala	GCC	1	0.1	His	CAU	86	1.74	Ser	AGA	19	1.3
Ala	GCU	20	2.05	Ile	AUC	61	0.37	Ser	AGC	3	0.21
Arg	CGA	4	1.23	Ile	AUU	267	1.63	Ser	AGU	13	0.89
Arg	CGG	1	0.31	Leu	CUA	73	0.62	Ser	UCA	37	2.53
Arg	CGU	8	2.46	Leu	CUC	37	0.32	Ser	UCC	1	0.07
Asn	AAC	19	0.24	Leu	CUG	39	0.33	Ser	UCU	44	3.01
Asn	AAU	139	1.76	Leu	CUU	120	1.03	Thr	ACA	18	1.2
Asp	GAC	16	0.26	Leu	UUA	340	2.91	Thr	ACC	2	0.13
Asp	GAU	107	1.74	Leu	UUG	92	0.79	Thr	ACU	40	2.67
Cys	UGC	1	0.08	Lys	AAA	93	1.51	Trp	UGA	24	1.85
Cys	UGU	24	1.92	Lys	AAG	30	0.49	Trp	UGG	2	0.15
Gln	CAA	95	1.3	Met	AUA	224	1.56	Tyr	UAC	67	0.37
Gln	CAG	51	0.7	Met	AUG	64	0.44	Tyr	UAU	292	1.63
Glu	GAA	92	1.22	Phe	UUC	54	0.28	Val	GUA	49	1.26
Glu	GAG	59	0.78	Phe	UUU	326	1.72	Val	GUC	11	0.28
Gly	GGA	35	2.8	Pro	CCA	9	0.97	Val	GUG	20	0.52
Gly	GGG	2	0.16	Pro	CCC	3	0.32	Val	GUU	75	1.94
Gly	GGU	13	1.04	Pro	CCG	3	0.32				

## References

[B12668562] Aguado-Aranda Pablo, Ricarte Antonio, Nedeljković Zorica, Hauser Martin, Kelso Scott, Sainz-Escudero Lucía, Skevington Jeffrey H., Marcos-García María Ángeles (2024). Unveiling the Mainland vs. insular variability of the Eumerus barbarus species group (Diptera: Syrphidae) in the western Mediterranean basin. Insects.

[B12668544] Ballester-Torres Iván, Ricarte Antonio, Nedeljković Zorica, Marcos-García M. Ángeles (2022). High phenotypic diversity does not always hide taxonomic diversity: A study case with Cheilosia soror (Zetterstedt, 1843) (Diptera: Syrphidae) in the Iberian Peninsula. Journal of Zoological Systematics and Evolutionary Research.

[B12339662] Barkalov A. V., Mutin V. A. (2018). Checklist of the hover-flies (Diptera, Syrphidae) of Russia. Euroasian Entomological Journal.

[B12339671] Bolger Anthony M., Lohse Marc, Usadel Bjoern (2014). Trimmomatic: a flexible trimmer for Illumina sequence data. Bioinformatics.

[B12339680] Cameron Stephen L. (2014). Insect mitochondrial genomics: Implications for evolution and phylogeny. Annual Review of Entomology.

[B12339689] Chen Mengchen, Peng Ke, Su Chengyong, Wang Yunliang, Hao Jiasheng (2021). The complete mitochondrial genome of *Syrphusribesii* (Diptera: Syrphoidea: Syrphidae). Mitochondrial DNA Part B.

[B12339699] Choi D. S., Suk S. W., Lee S. B., Han H. Y. (2018). Syrphidae III, Arthropoda: Insecta: Diptera: Brachycera: Syrphidae: Syrphinae. In: Insect Fauna of Korea.

[B12339707] Cook David F, Voss Sasha C, Finch Jonathan T D, Rader Romina C, Cook James M, Spurr Cameron J (2020). The role of flies as pollinators of horticultural crops: An Australian case study with worldwide relevance. Insects.

[B12339718] Cumming J., Wood D., Kirk-Spriggs A. H., Sinclair B. J. (2017). Manual of Afrotropical Diptera. Vol. 1. Introductory chapters and keys to Diptera families.

[B12339731] Dunn Lucinda, Lequerica Manuel, Reid Chris R, Latty Tanya (2020). Dual ecosystem services of syrphid flies (Diptera: Syrphidae): pollinators and biological control agents. Pest Management Science.

[B12339740] Dušek J., Láska P. (1967). Versuch zum Aufbau eines naturlichen Systems mitteleuropaischer Arten der Unterfamilie Syrphinae (Diptera). Acta Scientiarum Naturalium Academiae Scientiarum Bohemicae Brno.

[B12339749] Dušek J., Láska P. (1976). European species of *Metasyrphus*: key, descriptions and notes (Diptera, Syrphidae). Acta Entomologica Bohemoslovaca.

[B12339758] Evenhuis N. L. (2018). Nomenclatural studies toward a World List of Diptera genus-group names. Part VI: Daniel William Coquillett. Zootaxa.

[B12668500] Evenhuis N. L., Pape T. Systema Dipterorum, Version 5.3. http://www.diptera.org/.

[B12339767] Gilbert F. S. (1985). Size and shape variation in *Syrphusribesii* L. (Diptera, Syrphidae). Proceedings of the Royal Society of London. Series B. Biological sciences.

[B12339776] Greiner Stephan, Lehwark Pascal, Bock Ralph (2019). OrganellarGenomeDRAW (OGDRAW) version 1.3.1: expanded toolkit for the graphical visualization of organellar genomes. Nucleic Acids Research.

[B12339785] Han H. Y., Choi D. S. (2001). Family Syrphidae.

[B12339793] Han H. Y., Norrbom A. L. (2005). A systematic revision of the New World species of *Trypeta* Meigen (Diptera: Tephritidae). Systematic Entomology.

[B12339802] Han H. Y., Suk S. W., Lee Y. B., Lee H. S. (2014). National list of species of Korea, Insect (Diptera II).

[B12339810] Han H. Y., Kang H. J., Kim S. K., Kim W. K., Kim C. O., Byun H. W., Seo S. J., Euo S. S., Lee Y. B., Lee H. I., Choi J. H., Ham D. S., Park J. K. (2021). Check list of Insects from Korea.

[B12339831] Hodgkiss Dylan, Brown Mark J. F., Fountain Michelle T. (2018). Syrphine hoverflies are effective pollinators of commercial strawberry. Journal of Pollination Ecology.

[B12339840] Hua L. Z. (2006). List of Chinese insects. Vol. IV.

[B12339848] Huang C., Cheng X., Yang C., Xue W., Chao C. (1996). Flies of China.

[B12339861] Huo K. K., Guodong R., Zhemin Z. (2007). Fauna of Syrphidae from Mt. Qinling-Bashan in China (Insecta: Diptera).

[B12668453] Huo K. K., Yang D., Wang M. Q., Li W. L. (2020). Species catalogue of China. Vol. 2. Animals, Insecta (VII), Diptera (3):Cyclorrhaphous Brachycera..

[B12339869] Inouye David W, Larson Brendon M. H., Ssymank Axel, Kevan Peter G. (2015). Flies and flowers III: Ecology of foraging and pollination. Journal of Pollination Ecology.

[B12339878] Katoh K., Standley D. M. (2013). MAFFT Multiple Sequence Alignment Software Version 7: Improvements in Performance and Usability. Molecular Biology and Evolution.

[B12339887] Kim Chan-Ouk, Han Ho-Yeon (2022). Clarifying the identity of two resembling hoverfly species, *Betasyrphusserarius* and *B.nipponensis* (Diptera: Syrphidae: Syrphini), based on morphology and DNA barcoding. Journal of Asia-Pacific Entomology.

[B12339896] Li Hu, Yan Yan, Li Juan (2023). Eighteen mitochondrial genomes of Syrphidae (Insecta: Diptera: Brachycera) with a phylogenetic analysis of Muscomorpha.. PLOS One.

[B12339905] Liu H., Zhao L., Li G., He Y., Huo K. K. (2022). The complete mitochondrial genome of *Melanostomamellinum* (Linnaeus, 1758) (Diptera: Syrphidae) and phylogenetic analysis. Mitochondrial DNA Part B.

[B12339915] Li Xiaoli, Ding Shuangmei, Li Xin, Hou Peng, Tang Chufei, Yang Ding (2017). The complete mitochondrial genome analysis of *Eristalistenax* (Diptera, Syrphidae). Mitochondrial DNA Part B.

[B12339926] Mengual Ximo, Ståhls Gunilla, Rojo Santos (2008). First phylogeny of predatory flower flies (Diptera, Syrphidae, Syrphinae) using mitochondrial COI and nuclear 28S rRNA genes: conflict and congruence with the current tribal classification. Cladistics.

[B12339935] Mengual Ximo (2015). The systematic position and phylogenetic relationships of *Asiobaccha* Violovitsh (Diptera, Syrphidae). Journal of Asia-Pacific Entomology.

[B12339944] Mengual Ximo, Ståhls Gunilla, Láska Pavel, Mazánek Libor, Rojo Santos (2018). Molecular phylogenetics of the predatory lineage of flower flies *Eupeodes*-*Scaeva* (Diptera: Syrphidae), with the description of the Neotropical genus *Austroscaeva* gen. nov.. Journal of Zoological Systematics and Evolutionary Research.

[B12339954] Mengual Ximo (2020). Phylogenetic relationships of the bacchine flower flies (Diptera: Syrphidae) based on molecular characters, with a description of a new species of *Melanostoma* (Schiner, 1860). Contributions to Zoology.

[B12339963] Mengual Ximo, Mayer Christoph, Burt Trevor O., Moran Kevin M., Dietz Lars, Nottebrock Gaby, Pauli Thomas, Young Andrew D., Brasseur Marie V., Kukowka Sandra, Kelso Scott, Etzbauer Claudia, Bot Sander, Hauser Martin, Jordaens Kurt, Miranda Gil F. G., Ståhls Gunilla, van Steenis Wouter, Peters Ralph S., Skevington Jeffrey H. (2023). Systematics and evolution of predatory flower flies (Diptera: Syrphidae) based on exon‐capture sequencing. Systematic Entomology.

[B12339988] Mik Josef (1897). Einige Bemerkungen zur Dipteren-Familie der Syrphiden. Wiener Entomologische Zeitung.

[B12339997] Milankov VESNA, Ludoški JASMINA, Ståhls GUNILLA, Stameković JELENA, Vujić ANTE (2009). High molecular and phenotypic diversity in the *Merodonavidus* complex (Diptera, Syrphidae): Cryptic speciation in a diverse insect taxon. Zoological Journal of the Linnean Society.

[B12340007] Minh B. Q., Nguyen M. A. T., von Haeseler A. (2013). Ultrafast approximation for phylogenetic bootstrap. Molecular Biology and Evolution.

[B12340016] Moran Kevin M, Skevington Jeffrey H, Kelso Scott, Mengual Ximo, Jordaens Kurt, Young Andrew D, Ståhls Gunilla, Mutin Valerii, Bot Sander, van Zuijen Menno, Ichige Katsuyoshi, van Steenis Jeroen, Hauser Martin, van Steenis Wouter (2022). A multigene phylogeny of the eristaline flower flies (Diptera: Syrphidae), with emphasis on the subtribe Criorhinina. Zoological Journal of the Linnean Society.

[B12340035] Mutin V. A., Barkalov A. V., Lehr P. A. (1999). Key to the insects of Russian Far East. Vol. VI. Diptera and Siphonaptera. Pt 1.

[B12663662] Nguyen Lam-Tung, Schmidt Heiko A., von Haeseler Arndt, Minh Bui Quang (2015). IQ-TREE: a fast and effective stochastic algorithm for estimating maximum-likelihood phylogenies. Molecular Biology and Evolution.

[B12340057] Resources] NIBR [National Institute of Biological (2019). National Species list of Korea. III. Insects (Hexapoda).

[B12340065] Paek M. K., Hwang J. M., Jung K. S., Kim T. W., Kim M. C., Lee Y. J., Cho Y. B., Park S. W., Lee H. S., Ku D. S., Jeong J. C., Kim K. G., Choi D. S., Shin E. H., Hwang J. H., Lee J. S., Kim S. S., Bae Y. S., Paek M. K., Cho Y. K. (2010). Nature & Ecology Academics. Series 2.

[B12340092] Peck L. V., Soós Á., Papp L. (1988). Catalogue of Palaearctic Diptera.

[B12340105] Pu De-qiang, Liu Hong-ling, Gong Yi-yun, Ji Pei-cheng, Li Yue-jian, Mou Fang-sheng, Wei Shu-jun (2017). Mitochondrial genomes of the hoverflies *Episyrphusbalteatus* and *Eupeodescorollae* (Diptera: Syrphidae), with a phylogenetic analysis of Muscomorpha. Scientific Reports.

[B12340117] Ricarte Antonio, Souba-Dols Gabriel J., Skevington Jeffrey H., Quinto Javier, García Mª Ángeles Marcos (2020). Morphological, genetic and biological evidences to understand *Meromacrus* Rondani diversity: New species and early stages (Diptera: Syrphidae). Insects.

[B12340127] Rotheray G., Gilbert F. (1999). Phylogeny of Palaearctic Syrphidae (Diptera): evidence from larval stages. Zoological Journal of the Linnean Society.

[B12340136] Rotheray G. E., Gilbert F. (2011). The natural history of hoverflies.

[B12689667] Schiner Ignaz Rudolph (1861). Fauna Austriaca: die Fliegen (Diptera) Nach der analytischen Methode bearb.,mit der Characteristik almmilicher europäischer Gattungen, der Beechraibung aller in Deutschland vorkommenden Arten und der Aufzahlung aller bisher beschriebenen europaischen Arten. C. Gerolds Sohn.

[B12340157] Schwarz Gideon (1978). Estimating the Dimension of a Model. Annals of Statistics.

[B12340166] Shatalkin A. I. (1975). A taxonomic analysis of the hover flies (Diptera, Syrphidae). I. Entomological Review.

[B12340175] Shiraki T. (1930). Die Syrphiden des Japanischen Kaiserreichs, mit Berucksichtigung benachbarter Gebiete.

[B12340183] Sommaggio Daniele (1999). Syrphidae: can they be used as environmental bioindicators?. Agriculture, Ecosystems and Environment.

[B12340192] Ssymank Axel, Kearns C. A., Pape Thomas, Thompson F. Christian (2008). Pollinating flies (Diptera): A major contribution to plant diversity and agricultural production. Biodiversity.

[B12340201] Stephens J. F., Newman E. (1841). Entomological notes [part].

[B12340214] Tamura Koichiro, Stecher Glen, Kumar Sudhir (2021). MEGA11: Molecular Evolutionary Genetics Analysis Version 11.. Molecular biology and evolution.

[B12340223] Community The Galaxy (2024). The Galaxy platform for accessible, reproducible, and collaborative data analyses: 2024 update.. Nucleic acids research 2024; gkae410.

[B12340232] Thompson F. C., Rotheray G., Papp L., Darvas B. (1998). Manual of Palaearctic Diptera, Vol. 3.

[B12340245] Thompson F. C. (1999). A key to the genera of the flower flies (Diptera: Syrphidae) of the Neotropical Region including descriptions of new genera and species and a glossary of taxonomic terms. Contributions on Entomology, International.

[B12340254] Violovitsh N. A., Tsherepanov A. I. (1975). Taksonomyia i ecologiya zhivotnykh Sibiri (Novye I maloizvestnye vidy fauny Sibiri).

[B12340267] Violovitsh N. A. (1983). Sirfidy Sibiri (Diptera, Syrphidae).

[B12340275] Vockeroth J. R. (1969). A revision of the genera of the Syrphini (Diptera: Syrphidae). The Memoirs of the Entomological Society of Canada.

[B12340284] Wong Daniel, Norman Hannah, Creedy Thomas J, Jordaens Kurt, Moran Kevin M, Young Andrew, Mengual Ximo, Skevington Jeffrey H, Vogler Alfried P (2023). The phylogeny and evolutionary ecology of hoverflies (Diptera: Syrphidae) inferred from mitochondrial genomes.. Molecular phylogenetics and evolution.

